# The efficacy of *QingfengGanke* granule in treating postinfectious cough in pathogenic *wind* invading *lungs* syndrome: a multicenter, randomized, double-blind, placebo-controlled trial

**DOI:** 10.1186/s13020-015-0049-6

**Published:** 2015-08-09

**Authors:** Hongli Jiang, Bing Mao, Lei Wang, Ruiming Zhang, Bin She, Faguang Jin, Yanling Xu, Jian Ma, Qiuping Liu

**Affiliations:** Pneumology Group, Department of Integrated Traditional Chinese and Western Medicine, West China Hospital, Sichuan University, No. 37 Guoxue Street, Chengdu, 610041 Sichuan Province People’s Republic of China; Department of Respiratory Medicine, Tangdu Hospital, The Fourth Military Medical University, No. 1 Xinsi Road, Xi’an, 710038 Shaanxi Province People’s Republic of China; Department of Respiratory Medicine, The Affiliated Hospital to Liaoning University of Traditional Chinese Medicine, No. 33 Beiling Street, Shenyang, 110032 Liaoning Province People’s Republic of China; Department of Respiratory Medicine, Nanjing First Hospital, No. 68 Changle Road, Nanjing, 210006 Jiangsu Province People’s Republic of China; Department of Respiratory Medicine, Baotou Central Hospital, No. 61 Huangcheng Road, Baotou, 014025 Inner Mongolia People’s Republic of China

## Abstract

**Background:**

Postinfectious cough (PIC) significantly affects cough-related quality of life but still lacks effective treatments. This study aims to investigate the efficacy of *QingfengGanke* granule (QFGKG) in treating PIC induced by pathogenic *wind* invading *lungs* syndrome.

**Methods:**

A multicenter, randomized, double-blind, placebo-controlled clinical trial was conducted. A total of 180 eligible participants were randomly (1:1:1) assigned to group A (QFGKG 6 g plus QFGKG-matched placebo 6 g), group B (QFGKG 12 g), and group C (QFGKG-matched placebo 12 g). All herbal medications were orally administered twice daily for 10 consecutive days. The primary outcome was time to cough resolution, and secondary outcomes included time to cough alleviation, mean changes in cough symptom score (CSS), visual analogue scale (VAS) score, cough-specific quality of life questionnaire (CQLQ) score, and traditional Chinese medicine (TCM) syndrome score from baseline to Day 10, as well as adverse events.

**Results:**

A total of 173 participants were included in the efficacy and safety analyses (group A, n = 57; group B, n = 57; group C, n = 59). The median time to cough resolution in groups A, B, and C was more than 10 days, 8 days, and more than 10 days, respectively (*P* < 0.0001), and the median time to cough alleviation was 4, 4, and 6 days, respectively (*P* < 0.0001). Compared with the placebo condition, groups A and B showed significantly greater improvements in CSS (*P* = 0.0005, *P* < 0.0001, respectively), VAS (*P* = 0.0002, *P* < 0.0001, respectively), CQLQ (*P* = 0.0258, *P* = 0.0003, respectively), and TCM syndrome (*P* = 0.0031, *P* < 0.0001, respectively). The time to cough resolution was faster in group B compared with group A (*P* = 0.0091). The adverse event profiles were comparable among the three groups.

**Conclusion:**

*QingfengGanke* granule is efficacious in the treatment of PIC induced by pathogenic *wind* invading *lungs* syndrome.

**Electronic supplementary material:**

The online version of this article (doi:10.1186/s13020-015-0049-6) contains supplementary material, which is available to authorized users.

## Background

Postinfectious cough (PIC), a cough lasting for at least 3 weeks but not more than 8 weeks, is the most common cause of subacute cough, accounting for almost 50% of cases [1]. Respiratory viruses including influenza, *Mycoplasma pneumoniae*, *Chlamydia pneumoniae*, *Moraxella catarrhalis*, and *Bordetella pertussis* have been implicated as causes of PIC [2–6]. PIC occurs in approximately 10–40% of patients with upper respiratory tract infection (URTI) [2, 3]. During outbreaks of influenza or atypical infections, PIC may affect 25–50% of cases [2, 4–6].

According to traditional Chinese medicine (TCM) theory, *qi* is managed mainly by the *lungs*, which control inspiration and expiration, and is particularly susceptible to pathogenic *wind*, leading to failure of the *lungs* to disperse and descend. This results in dysregulation of dispersing and descending *lung qi*, manifested as coughing symptoms. PIC may be classified into the TCM disease category of “exogenous cough”; clinical practice and TCM syndrome research indicates that the “pathogenic *wind* (*feng*) invading *lungs* (*fei*)” syndrome is the most common type, which is mainly manifested as cough, throat itchiness, throat dryness, chest tightness, and expectoration, along with pale red tongue, white or yellow tongue coating, and floating pulse [7].

Several pharmaceutical drugs have been used to relieve PIC, including inhaled ipratropium bromide, central acting antitussive agents such as codeine and dextromethorphan, and inhaled or oral corticosteroids; however, drugs with optimal clinical efficacy and safety profiles are not yet available in Western medicine [8]. Evidence from randomized controlled trials (RCTs) of Chinese herbal medicine (CHM) treatments of PIC is not yet convincing because of small sample sizes and poor reporting quality [9].

*QingfengGanke* granule (QFGKG) consists of *Mahuang* (*Ephedra sinica* Stapf.), *Qingfengteng* (*Sabia japonica* Maxim.), *Baibu* (*Stemona japonica* (Bl.) Miq.), and *Ziwan* (*Aster tataricus* L. f.), with starch and sucrose excipients. The sources, pharmacological actions, and dosage of each ingredient are listed in Table 1. One preclinical pharmacodynamic experiment indicated the anti-inflammatory and immune modulatory properties of GFGKG (unpublished observation by Jia Jinliang), and preliminary clinical observational studies have shown its potential to relieve cough [10]. In addition, acute and chronic toxicological studies have revealed that GFGKG has no apparent toxic side effects (unpublished observation by Jia Jinliang).

**Table 1:** QFGKG formula

Chinese Pinyin name	Botanical authority	Family name	Source	Pharmacological actions in TCM	Dosage/proportion (%) crude herb/1 g of QFGKG
*Mahuang*	*Ephedra sinica Stapf.*	Ephedraceae	Dried herbaceous stem	To release the exterior, disperse *wind*-*cold*; to stop wheezing due to obstruction of *lung*-*qi*; to reduce edema and promote urination especially due to obstruction from external *wind*-*cold* pathogen	375 mg/23.08
*Qingfengteng*	*Sabia japonica Maxim.*	Sabiaceae	Dried rattan	To expel *wind*-*damp*; to promote urination; to dredge collaterals or channels	500 mg/30.76
*Baibu*	*Stemona japonica (Bl.) Miq.*	Stemonaceae	Dried tuberous root	To moisten the *lungs* and relieve cough, excellent for chronic respiratory disorders and chronic cough; anti-parasitic	375 mg/23.08
*Ziwan*	*Aster tataricus L. f.*	Compositae	Dried root and rhizome	To moisten the *lungs* and descend *lung*-*qi*; to stop acute or chronic cough	375 mg/23.08

*QFGKG* *QingfengGanke* granule, *TCM* traditional Chinese medicine.

This study aims to investigate the efficacy of QFGKG in treating PIC induced by pathogenic *wind* invading *lungs* syndrome.

## Methods

### Study design

Written informed consent was obtained from each subject prior to participation (Additional file 1). The study protocol was reviewed and approved by the Medical Ethics Committee of West China Hospital at Sichuan University (Chengdu, China) (Additional files 2, 3, respectively). This study was conducted in accordance with the Declaration of Helsinki and Good Clinical Practice ethical guidelines [11]. In addition, the trial was authorized by the China State Food and Drug Administration (No. 2010L00279) and registered in the Chinese Clinical Trial Registry (ChiCTRTRC12002297). All herbs used in this trial are generally regarded as safe for use according to the China State Food and Drug Administration. The trial was reported in accordance with the Consolidated Standards of Reporting Trials (CONSORT) statement (Additional file 4) [12].

The randomization sequence was computer generated in blocks of five in a 1:1:1 ratio by an independent statistician using SAS statistical software (SAS 9.3, SAS Institute, USA). Allocation details were stored in two sealed envelopes. One set of envelopes was kept by QiHuang Drug Clinical Research Center (Beijing, China) and the other set was kept by the institutions at which the study was conducted to be opened in case of emergency. All researchers, participants, and statisticians were masked to treatment allocation throughout the study.

### Setting and participants

Potential participants diagnosed with PIC induced by pathogenic *wind* invading *lungs* syndrome, aged 18–65 years old, were recruited by respiratory specialists with more than 5 years’ working experience from the outpatient departments of five participating centers across China: West China Hospital of Sichuan University, Tangdu Hospital of the Fourth Military Medical University, the Affiliated Hospital to Liaoning University of TCM, Nanjing First Hospital, and Baotou Central Hospital. PIC was defined as a persistent cough triggered by URTI and lasting for 3–8 weeks [8, 13]. Using the Chinese national criteria for clinical diagnosis and treatment evaluation of cough [14], pathogenic *wind* invading *lungs* syndrome was diagnosed in the presence of cough, throat itchiness, cough aggravated by pathogenic *wind*, and at least one of the other minor symptoms, along with pale red tongue, white or yellow tongue coating, and floating pulse. All participant inclusion and exclusion criteria are listed in Table 2.

**Table 2:** Inclusion and exclusion criteria

Inclusion criteria
Clinical diagnosis of PIC
Pathogenic-*wind* invading *lungs* syndrome in TCM
Baseline CSS of 2 or greater in the daytime and/or nighttime
Cough lasting for at least 3 weeks but not more than 8 weeks
Aged 18–65 years old
Participant must voluntarily give written informed consent and report adverse events
Exclusion criteria
Wheezing or rales on physical examination
Bronchial provocation test positive
Abnormal findings on plain chest radiograph
Use of an angiotensin-converting-enzyme inhibitor within the past 2 months
Presenting with heartburn or regurgitation
Symptoms or signs of upper airway cough syndrome
Current smoker or recent ex-smoker (giving up smoking for less than 3 months)
Participant has severe primary disease of pulmonary, hepatic, renal, or hematological system, or other serious diseases affected the survival, such as cancer or AIDS
ALT or AST >1.5 times of normal upper limit, abnormal serum creatine, abnormal serum Ig E, eosinophilia in the blood, white blood cell count <3× 10^9^/L or >10 × 10^9^/L/L, and/or neutrophil granulocyte >80%
Pregnancy or lactating women, or women who have birth plan
Allergic constitution or known to be allergic to any component in QFGKG
Participants taking similar medicines in the last 1 month or having participated or participating in the other trials in last 3 months

*CSS* cough symptom score, *PIC* postinfectious cough, *QFGKG* *QingfengGanke* granule, *TCM* traditional Chinese medicine.

### Intervention

All participants were randomly allocated in a double-blind manner to one of three parallel treatment groups: group A received QFGKG 6 g plus QFGKG-matched placebo 6 g, twice daily; group B took orally QFGKG 12 g, twice daily; and group C took orally QFGKG-matched placebo 12 g, twice daily. The scheduled treatment duration was 10 days and the participants were followed up by scheduling visits to the clinic at baseline (Day 0), Day 5, and Day 10. All participants were treated in an equivalent fashion and instructed to avoid taking any other medication for the relief of cough. Compliance was checked by examining a daily diary card and returned medication at the end of the study. Active and placebo granules that were indistinguishable in taste, color, appearance, and smell were issued in a sealed box and supplied by Baotou Chinese Traditional Medicine Co., Ltd. (China). All medications were dispensed by a specified drug administrator in a separate reception room.

### Outcome measures

#### Cough symptom score (CSS)

The CSS is a validated tool to evaluate the frequency and severity of cough. Participants provided self-rated CSSs ranging from 0 to 3. Each evaluation day was divided into two intervals: daytime (from 8:00 am to 8:00 pm) and nighttime (from 8:00 pm to 8:00 am) [13]. CSS was evaluated every day during the study.

#### Visual analogue scale (VAS)

The VAS is a 10 cm continuous horizontal line that measures the severity of cough. Participants were instructed to rate their cough severity from “no cough” to “extremely strong cough.” Scores ranged from 0 to 10. VAS score was assessed at baseline (Day 0), Day 5, and Day 10.

#### Cough-specific quality of life questionnaire (CQLQ)

The CQLQ was designed specifically to evaluate decrements in disease-targeted quality of life caused by cough and consists of 28 items in three domains (physical, social, and psychological) [15]. A recent systematic review [16] showed that the CQLQ can provide valid and reliable outcomes for cough research. The minimum and maximum CQLQ scores were 28 and 112, respectively, with lower scores indicating a better quality of life. CQLQ score was assessed at baseline and Day 10.

### TCM syndrome score

Using the Chinese national criteria for clinical diagnosis and treatment evaluation of cough [14], the main and minor symptoms of the TCM syndrome were graded (cough: not at all = 0, mild = 2, moderate = 4, severe = 6; throat itchiness, throat dryness, and chest tightness: not at all = 0, mild = 1, moderate = 2, severe = 3; cough aggravated by pathogenic *wind* and expectoration: yes = 0 and no = 2). The TCM syndrome score was the accumulated score of the main symptom score and all minor symptom scores. Additionally, tongue proper, tongue coating, and pulse were also assessed but not scored. TCM syndrome score was assessed at baseline and Day 10.

### Safety evaluation

Safety evaluations included monitoring of adverse events (AEs), as well as clinical and laboratory findings. Participants were required to record any unexpected symptoms, signs, or feelings during the treatment period. In addition, routine tests of blood, urine and stool, hepatic and renal functions, and electrocardiogram were also performed at baseline and again after the 10-day treatment to assess safety for each group.

The primary outcome was mean or median time to cough resolution, which was assessed as the length of time from the start of treatment until the first point at which cough resolved. Cough resolution was defined as both daytime and nighttime CSS decreasing to 0. The secondary outcomes included time to cough alleviation, mean changes in CSS, VAS score, CQLQ score, and TCM syndrome score from baseline to Day 10, as well as adverse events. Cough alleviation was defined as a reduction in both daytime and nighttime CSS ≥ 1, lasting for 2 consecutive days.

### Sample size determination

No clinical trial data existed on the efficacy of QFGKG. Based on our experience and previous studies of other CHM formulae used to treat PIC [17–19], the cough resolution rates in the CHM and placebo groups were estimated to be 40 and 15%, respectively. The sample size for each group was calculated to be 53 according to the formula n = (U_α_ + U_β_)^2^ × 2P × (1 − P)/(P_1_ − P_0_)^2^ with a power of 90% or greater, assuming a significance level of 0.05. To allow for a dropout rate of approximately 10%, 180 PIC participants were needed for this study.

### Statistical analysis

All the efficacy and safety analyses were performed using the intent-to-treat population. All data were documented by two independent individuals and the data files were secured. Statistical analyses were conducted using statistical software (SAS 9.3, SAS Institute, USA). Normally distributed quantitative data were described as mean ± standard deviation and analyzed using analysis of variance. Quantitative data that were not normally distributed were expressed as median and analyzed by the Wilcoxon rank-sum test. Qualitative data were presented as frequency and percentage and analyzed using Chi square (χ^2^) or Fisher’s exact test. The primary outcome was analyzed using the survival method. Kaplan–Meier curves were constructed and differences between curves were tested by the log-rank method. The secondary outcomes were analyzed using analysis of covariance with a Bonferroni correction, or the survival method. A two-tailed *P* value of less than 0.05 was considered statistically significant.

## Results

### Participant characteristics

**Figure 1:**
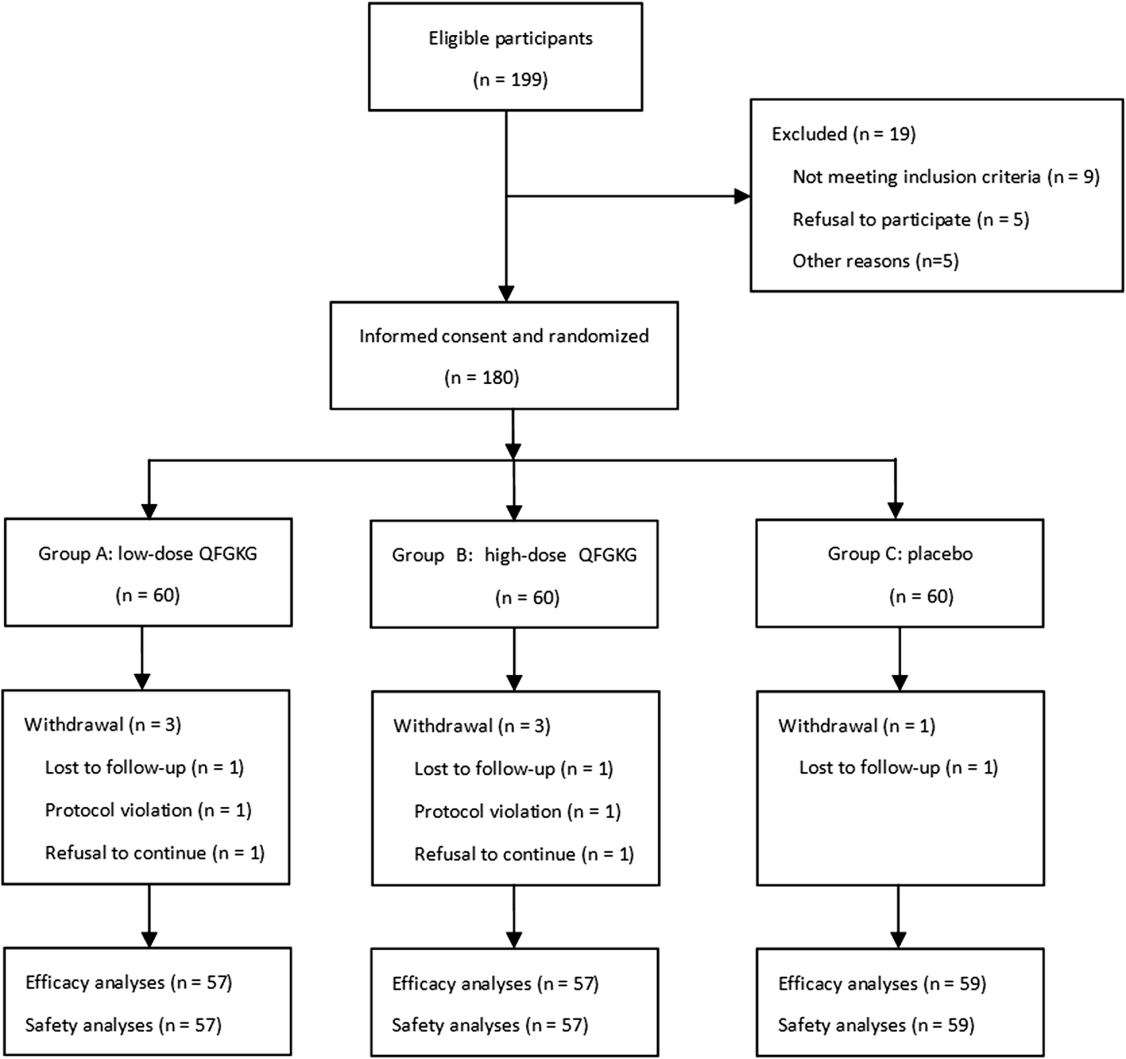
Flow chart of participants through study.

A total of 180 eligible participants were enrolled from five tertiary hospitals across China between April 2011 and March 2012, and randomly assigned 1:1:1 to receive low-dose QFGKG (group A, n = 60), high-dose QFGKG (group B, n = 60), or placebo (group C, n = 60). Of these, seven participants were withdrawn from the study for several reasons, including protocol violation (group A, n = 1; group B, n = 1), lost to follow-up (group A, n = 1; group B, n = 1; group C, n = 1), and refusal to continue (group A, n = 1; group B, n = 1). Overall, 173 participants were included in the efficacy and safety analyses (Figure 1). Compliance measured by daily diary card and returned medication was 96%. Use of concomitant medications including herbal and complementary medicines did not differ across the three groups (*P* = 0.9378). No differences in demographic or clinical characteristics were observed among the three groups at baseline (Table 3).

**Table 3:** Demographic and clinical characteristics at baseline

Characteristics	Group A (n = 57)	Group B (n = 57)	Group C (n = 59)	*P* values*
Age (years)	44.37 ± 12.38	43.07 ± 12.67	44.07 ± 12.65	0.8663
Gender (female/%)	35 (61.40)	35 (61.40)	31 (52.54)	0.5336
Body weight (kg)	62.41 ± 9.91	62.45 ± 10.76	63.44 ± 10.87	0.6680
Height (cm)	164.26 ± 7.89	163.22 ± 7.99	164.47 ± 8.59	0.6097
Former smoker (n (%))	10 (17.54)	5 (8.77)	5 (8.47)	0.2255
Cough duration (days)	33.00 ± 10.32	29.96 ± 7.09	30.80 ± 8.96	0.3685
Previous diagnosis of PIC (n (%))	9 (15.79)	8 (14.04)	10 (16.95)	0.9696
VAS score	6.04 ± 1.44	5.90 ± 1.23	5.96 ± 1.33	0.8341
CSS	3.84 ± 0.80	3.79 ± 0.77	3.86 ± 0.84	0.9648
Daytime CSS	2.12 ± 0.38	2.02 ± 0.35	2.14 ± 0.43	0.2084
Nighttime CSS	1.72 ± 0.65	1.77 ± 0.68	1.73 ± 0.69	0.7752
CQLQ score	64.51 ± 9.58	64.95 ± 7.31	64.78 ± 6.46	0.9068
TCM syndrome score	12.02 ± 2.60	11.46 ± 1.97	11.97 ± 2.17	0.1492

Values are reported as mean ± SD or as frequency (%).

*CQLQ* cough-specific quality of life questionnaire, *CSS* cough symptom score, *PIC* postinfectious cough, *TCM* traditional Chinese medicine, *VAS* visual analogue scale.

* P values obtained via variance analysis for continuous variables, or Chi square tests for categorical variables.

### Time to cough resolution

**Figure 2:**
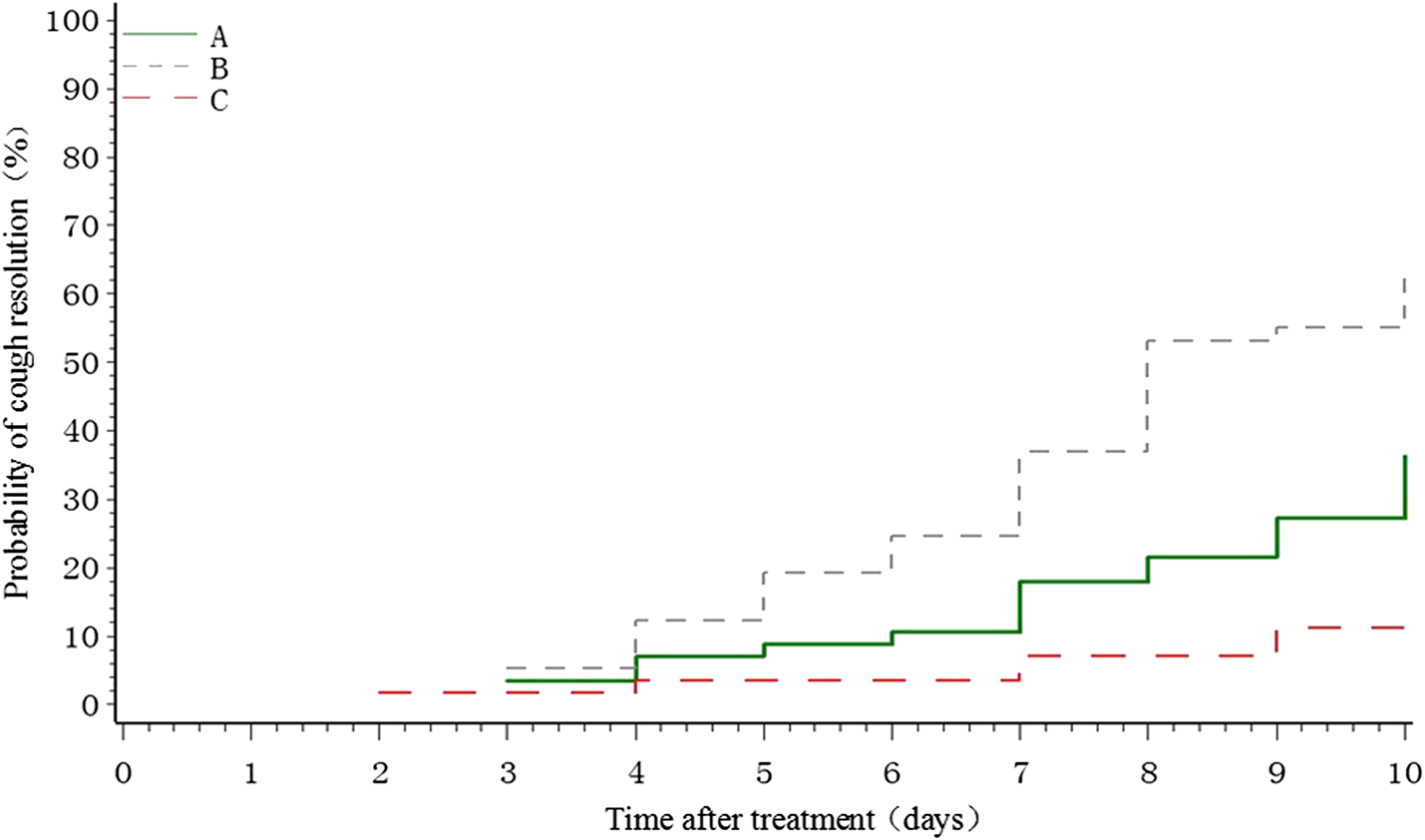
Time to cough resolution in participants with PIC.

The median time to cough resolution in groups A, B, and C was more than 10 days, 8 days, and more than 10 days, respectively. A significant therapeutic effect was noted in both QFGKG groups compared with the placebo condition (group A, *P* < 0.0001; group B, *P* < 0.0001), and the time to cough resolution was faster in group B compared with group A (*P* = 0.0091) (Figure 2).

### Time to cough alleviation

**Figure 3:**
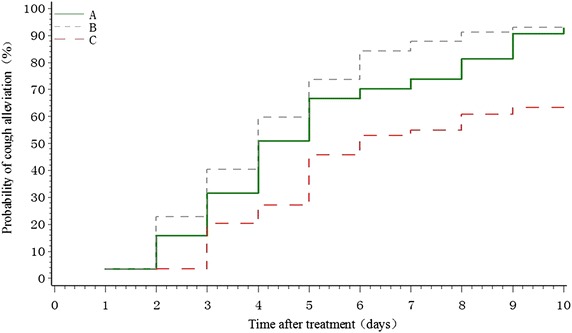
Time to cough alleviation in participants with PIC.

The median time to cough alleviation in groups A, B, and C was 4, 4, and 6 days, respectively. The between-group differences were statistically significant (group A, *P* = 0.0003; group B, *P* < 0.0001), except for the difference between group A and group B (*P* = 0.1843) (Figure 3).

### CSS

Significant improvements in CSS (both daytime and nighttime) were observed from baseline to Day 10 in all three groups (group A, 3.84 ± 0.80 to 1.14 ± 1.11; group B, 3.79 ± 0.77 to 0.79 ± 1.19; group C, 3.86 ± 0.84 to 2.22 ± 1.54). Both QFGKG groups showed significantly greater improvements compared with the placebo [group A, −1.07, 95% confidence interval (CI) −1.55 to −0.6, *P* = 0.0005; group B, −1.41, 95% CI −1.89 to −0.94, *P* < 0.0001]. Group B showed a greater, though non-significant, improvement compared with group A (*P* = 0.5406). Similar improvements were observed in both daytime and nighttime CSS (Table 4).

**Table 4:** Analyses of CSS, VAS, CQLQ scores, and TCM syndrome score

Outcome variables	Change from baseline to Day 10			
	Group A (n = 57)	Group B (n = 57)	Group C (n = 59)	*P* values*
CSS	−2.70 ± 1.30*	−3.00 ± 1.28*	−1.64 ± 1.64	<0.0001
Daytime CSS	−1.39 ± 0.70*	−1.58 ± 0.71*	−0.78 ± 0.87	<0.0001
Nighttime CSS	−1.32 ± 0.83*	−1.42 ± 0.75*	−0.86 ± 0.96	<0.0001
VAS	−4.24 ± 2.10*	−4.78 ± 2.01*	−2.55 ± 2.28	<0.0001
CQLQ score	−16.09 ± 14.14*	−20.12 ± 14.61*	−9.75 ± 11.99	<0.0001
TCM syndrome score	−8.53 ± 3.74*	−9.46 ± 3.00*	−5.80 ± 4.51	<0.0001

Values are reported as mean ± SD.

*CQLQ* cough-specific quality of life questionnaire, *CSS* cough symptom score, *TCM* traditional Chinese medicine, *VAS* visual analogue scale.

* *P* < 0.01 for group A/group B vs. group C.

### VAS

Significant changes from baseline in VAS score were found in all three groups (group A, 6.04 ± 1.44 to 1.81 ± 1.87; group B, 5.90 ± 1.23 to 1.12 ± 1.70; group C, 5.96 ± 1.33 to 3.41 ± 2.11). Both QFGKG groups showed significantly greater improvements compared with the placebo condition (group A, −1.63, 95% CI −2.31 to −0.95, *P* = 0.0002; group B, −2.26, 95% CI −2.95 to −1.58, *P* < 0.0001). Group B showed a greater, though non-significant, improvement compared with group A (*P* = 0.4200) (Table 4).

### CQLQ

Significant improvements in the total CQLQ scores were observed from baseline to Day 10 in all three groups (group A, 64.51 ± 9.58 to 48.27 ± 14.30; group B, 64.95 ± 7.31 to 44.82 ± 14.10; group C, 64.78 ± 6.46 to 54.89 ± 10.59). Compared with the placebo condition, participants in either QFGKG group had a better quality of life (group A, −6.17, 95% CI −10.4 to −1.88, *P* = 0.0258; group B, −9.65, 95% CI −13.9 to −5.4, *P* = 0.0003), but there was no significant difference between the high- and low-dose QFGKG groups (*P* = 0.5720) (Table 4).

### TCM syndrome score

Again, improvements in the total TCM syndrome score were observed from baseline to Day 10 in all three groups (group A, 12.02 ± 2.60 to 3.49 ± 3.51; group B, 11.46 ± 1.97 to 2.00 ± 3.00; group C, 11.97 ± 2.17 to 6.17 ± 4.00). Both QFGKG groups showed significantly greater improvements compared with the placebo condition (group A, −2.7, 95% CI −3.97 to −1.42, *P* = 0.0031; group B, −4.05, 95% CI −5.33 to −2.76, *P* < 0.0001), but the difference between the two QFGKG groups did not reach significance (*P* = 0.3692) (Table 4).

### Safety

There were neither serious AEs nor participant withdrawal because of AEs during the study. Participants taking QFGKG regardless of dosage reported more AEs than those in the placebo group (7.02 vs. 7.02 vs. 1.69%; *P* = 0.3313). In group A, AEs were reported in four participants. One participant reported dizziness and a rough tongue (at Day 1), which were resolved within 10 days. One participant complained of arm itchiness (at Day 1), which was resolved within 4 days. Another two reproductive-aged women showed a slightly elevated level of white blood cells in routine urine tests after treatment without any symptoms of urinary tract infection at Day 7 and 10, respectively; the events were resolved after drinking more water. In group B, four participants reported AEs. One male participant had mild hepatic dysfunction with baseline alanine aminotransferase 48 U/L increasing to 60 U/L after treatment, but dropping to normal when measured again 3 days later without any intervention. One participant developed URTI with an elevated level of white blood cells (7.31 × 10^9^/L pretreatment to 11.17 × 10^9^/L posttreatment); the event was resolved 7 days later after the administration of antibiotics. Two reproductive-aged women experienced an elevated level of white blood cells in routine urine tests after treatment without any discomfort at Day 6 and 11, respectively; the events were resolved after drinking more water. In group C, one participant complained of a mild episode of dizziness. All the reported AEs were generally considered mild in severity, manageable, and reversible.

## Discussion

To our knowledge, this is the first randomized, double-blind, placebo-controlled clinical trial of CHM in the treatment of PIC induced by pathogenic *wind* invading *lungs* syndrome. QFGKG could shorten the duration of cough, decrease cough severity and frequency, and improve cough-specific quality of life. In addition, this study found QFGKG to be safe and well tolerated. High-dose QFGKG appeared to be superior to low-dose QFGKG in shortening the duration of cough.

*QingfengGanke* granule improved cough and cough-related quality of life in both QFGKG groups. Similarly, significant improvements were found in the placebo group, most likely because of the self-limiting nature of PIC. In addition to natural recovery, the placebo effects might encompass several other components: regression of cough response toward the mean, true placebo effect, voluntary control, and effects related to expectancy and meaning of the treatment [20].

Although the pathogenesis of PIC remains unknown, airway inflammation, epithelial disruption, and cough hyper-responsiveness are involved [8]. The resurgence of pertussis or whooping cough has prompted wide public and clinical concerns over the last 20 years [21–23]. There is an increasing recognition of the presence of *Bordetella pertussis* infection in PIC, although URTI is the most common cause. Approximately 12–50% of cases with cough duration of 2 weeks or more show evidence of recent *B. pertussis* infection despite aggressive vaccination [24]. Although some medications are available, there is no proven effective therapy for patients with prolonged cough attributed to pertussis [25]. A recent double-blind, placebo-controlled RCT [26] indicated that nebulized ipratropium bromide in combination with salbutamol could reduce PIC. Another recent placebo-controlled RCT [27] showed that montelukast, a cysteinyl leukotriene type 1 receptor antagonist, was not an effective treatment for PIC. Whether there is a potential therapeutic effect of QFGKG on pertussis-related cough deserves further study.

In terms of safety, no deaths or serious AEs were reported; all AEs reported were of mild severity. Three AEs were categorized as possibly or probably related to the study medication: dizziness, rough tongue, and arm itchiness. An elevated level of white blood cells in routine urine tests, which occurred in four female participants in the QFGKG groups, was considered unrelated to the study medication and probably associated with an increased risk of urinary tract infection in young women of reproductive age. Some mild abnormalities observed in laboratory safety evaluations in the high-dose QFGKG group were judged as unrelated to the study medication. However, *Mahuang* in QFGKG may have some side effects, such as increased blood pressure, cardiac arrhythmia, excitability, insomnia, nausea, poor appetite, or convulsions [28].

Some potential limitations of the present study should be considered. First, cough frequency was subjectively assessed in this study using a cough diary. There is increasing evidence that acoustic cough-monitoring devices can be valid and reliable instruments with which to assess cough frequency. However, the absence of good responsiveness and inconsistency with other instruments has limited their wide use in cough research [16, 29]. Second, our study protocol specifically excluded elderly people and those with mild cough, limiting the generalizability of the study findings in real-life clinical practice. Third, there was no follow-up after treatment, so the effect of QFGKG on relapse rate after treatment was unknown. Moreover, the accuracy of determining the time to cough alleviation might have been impaired, as 2 days was needed to define cough alleviation.

## Conclusion

*QingfengGanke* granule is efficacious in the treatment of PIC induced by pathogenic *wind* invading *lungs* syndrome.

## Additional files

Additional file 1. Informed consent. Additional file 2. Study protocol. Additional file 3. Approval of ethics committee. Additional file 4. CONSORT checklist.

## Abbreviations

AE: adverse events
CHM: Chinese herbal medicine
CI: confidence interval
CONSORT: consolidated standards of reporting trials
CQLQ: cough-specific quality of life questionnaire
CSS: cough symptom score
PIC: postinfectious cough
QFGKG: *QingfengGanke* granule
RCT: randomized controlled trial
TCM: traditional Chinese medicine
URTI: upper respiratory tract infection
VAS: visual analogue scale
