# Supplementary material for: The efficacy of QingfengGanke granule in treating postinfectious cough in pathogenic wind invading lungs syndrome: a multicenter, randomized, double-blind, placebo-controlled trial
Source: Chin Med. 2015 Aug 9;10:21. doi: 10.1186/s13020-015-0049-6 (PMC4529711; doi:10.1186/s13020-015-0049-6)
Supplement: Additional file 1. — Informed consent. [file 13020_2015_49_MOESM1_ESM.pdf]

清风感咳颗粒临床研究受试者须知

尊敬的受试者：

经你的医生检查，你已确诊为感染后咳嗽（风邪恋肺证），我们将邀请你参加清风感咳颗粒治疗感染后咳嗽（风邪恋肺证）的临床研究。该药物是包头中药有限责任公司研制的中药新药，已获得国家食品药品监督管理局临床研究批件（批件号：2010L00279）。此临床研究是对清风感咳颗粒治疗感染后咳嗽（风邪恋肺证）有效性和安全性作出初步评价。

1.研究背景

清风感咳颗粒是在多年临床经验基础上总结出的用之于感染后咳嗽的临床有效方药，由麻黄、青风藤、百部、紫菀组成，功能主治为宣肺祛风、止咳化痰。用于风邪恋肺证，症见咳嗽、咽痒、痰黏而少、咽干、咳嗽常因吸入冷热空气或刺激性气味诱发加重、胸闷，感染后咳嗽见上述症侯者。

临床前药效学实验显示，清风感咳颗粒具有镇咳、祛痰、平喘、抗炎和免疫功能抑制作用。动物急毒、长毒实验研究显示，清风感咳颗粒按临床推荐剂量使用，毒副作用小，是安全可靠的，同时也不引起延迟性毒副反应。

按照国家中药新药临床研究指导原则的临床试验要求，受包头中药有限责任公司和北京岐黄药品临床研究中心委托，由四川大学华西医院为负责单位，另外四家医院为参加单位，对清风感咳颗粒治疗感染后咳嗽（风邪恋肺证）有效性和安全性作初步评价。

2.研究概述

采用区组随机对照，多中心临床试验方法，由四川大学华西医院负责与其它四所医院（辽宁中医药大学附属医院、南京市第一医院、包头市中心医院、第四军医大学唐都医院）协同完成。本临床研究预计有 180 例受试者自愿参加，试验药物为清风感咳颗粒，对照药物为清风感咳颗粒安慰剂。如果您符合入选标准并愿意参加本研究，您将被随机分配到试验组或对照组的任一组，服药方法为：清风感咳颗粒 6g/次+清风感咳颗粒安慰剂 6g/次，2 次/日，温水冲服；或清风感咳颗粒 12g /次，2 次/日，温水冲服；或清风感咳颗粒安慰剂 12g /次，2 次/日，温水冲服。试验时间为 10 天。

试验前研究者将完成您的病例登记、体格检查、心电图、血常规、小便常规、肝肾功能、胸片、支气管激发或舒张试验等各项实验室指标检查，还需耽误您几分钟时间完善 CQLQ 量表。服药后第 6 天、第 11 天观察记录一般情况、临床症状和体征。请您在服药结束后 1 天内，空腹来医院复查。医生将对您疾病的改善及实验室检测指标进行检查。研究期间请您按照医生的要求服药，按时复诊，并随时告知医生您的不适感受。研究期间，请您保证不服用其他治疗咳嗽的药物，如有其他合并用药请在访视时告知医生。

3. 不良反应

动物急毒、长毒实验研究显示，按临床推荐剂量使用，毒副作用小，是安全可靠的，同时也不引起延迟性毒副反应。

4. 可能的受益和风险

自愿参加本研究，你将得到免费的药物（本试验仅供 10 天，试验结束后不再供药）及相关的实验室检查，将可能使您的疾病得到改善。试验结束后，你还将获得申办方提供的交通补贴。

临床前实验研究虽未发现明显毒副反应，但你亦有可能会出现不可预知的不良反应。我们将认真检测你有可能发生的不良反应，如果在临床试验期间，出现因药物引起的已知的或目前未知的不良反应，医生均会给予你积极的治疗，申办单位将会负责由此引起的相关治疗费用。如果发生与受试药物相关的严重不良事件，除医生会给予积极治疗外，申办单位将会承担治疗费用和相应的赔偿。

5. 保密

本临床试验的结果只用于科研目的，因此您参加试验及试验中您的个人资料均属保密，将依照法律规定得到保护，不会泄露您的名字和身份，您的姓名不会出现在任何研究报告和公开出版物中。但国家食品药品监督管理局、华西医院伦理委员会、研究者、申办者或监察员等如因工作需要，按规定有权接触您所有的试验资料，包括临床试验观察表、实验数据等。

6. 自愿参加

本临床试验遵循《药物临床试验质量管理规范》和《赫尔辛基宣言》，并获得临床负责单位四川大学华西医院伦理委员会的审核、批准，方案设计合乎伦理要求，这将会保证您的权益在本试验中不受侵犯。

您参加本临床试验完全是自愿的，您可以拒绝参加或在任何时间退出试验，而不会遭到歧视或报复，您的医疗待遇与权益也不会受影响。如果您退出临床试验，为了安全考虑，您在退出时应该完成一些相应的医学检查。另外，如果研究者认为您不适合继续参加时，为保护您的利益，研究者可以决定您是否继续参加临床试验。退出试验的受试者会得到研究者有关下一步治疗的建议。

清风感咳颗粒临床试验受试者知情同意

本人已仔细阅读**“清风感咳颗粒临床研究受试者须知”**，已了解这是一项治疗感染后咳嗽（风邪恋肺证）药物有效性与安全性初步评价的临床研究，临床试验研究者已就此药的特点和可能存在的不良反应向我做了详细解释，并对有关问题给予了解答。我在充分了解受试者须知的全部内容以及参加受试带来的利益与风险后，自愿参加本试验。我已充分理解：

1、此项研究已经获得国家食品药品监督管理局批准（批件号：2010L00279）；

2、作为受试者，我将遵守本研究受试者须知要求；

3、本临床试验的结果用于科研目的，除外国家食品药品监督管理局、华西医院伦理委员会或申办单位等，我参加试验及试验中的个人资料均属保密，将依照法律规定得到保护；

4、我自愿参加本研究，如果在临床试验期间，出现因药物引起的已知的或目前未知的不良反应（包括发生与受试药物相关的严重不良事件），医生均会给予我积极的治疗，申办单位将会负责由此引起的相关治疗费用。如发生与受试药物相关的严重不良事件，我还会得到申办单位的相应赔偿；

5、我参加本临床试验完全是自愿的，我可以拒绝参加或在任何时间退出试验，而不会遭到歧视或报复，我的医疗待遇与权益亦不会受影响；

6、我自愿参加本试验，并与研究人员充分合作；

7、我保证如实、客观地向研究人员提供参加本研究前的健康状况及相关情况。

受试者签名：____________________　　研究者签名：____________________

联系电话：______________________　　联系电话：______________________

日期：______年______月______日　　日期：_______年______月_______日
