# Supplementary material for: The efficacy of QingfengGanke granule in treating postinfectious cough in pathogenic wind invading lungs syndrome: a multicenter, randomized, double-blind, placebo-controlled trial
Source: Chin Med. 2015 Aug 9;10:21. doi: 10.1186/s13020-015-0049-6 (PMC4529711; doi:10.1186/s13020-015-0049-6)
Supplement: Additional file 2. — Study protocol. [file 13020_2015_49_MOESM2_ESM.pdf]

清风感咳颗粒 II 期临床试验方案

清风感咳颗粒治疗感染后咳嗽（风邪恋肺证）有效性和安全性的随机、双盲、剂量平行对照、多中心 II 期临床试验

临床试验批件号：	2010L00279
文件类型：	临床试验方案
研发阶段：	Ⅱ期临床
申办者：	包头中药有限责任公司
临床研究负责单位：	四川大学华西医院
方案设计者：	毛兵　　主任医师
方案审核者：	张瑞明　主任医师
统计单位：	北京岐黄药品临床研究中心
计划开始日期：	2011 年 2 月
计划完成日期：	2012 年 2 月
版本号	第 3 版
版本日期：	2011 年 01 月

内容保密

未经四川大学华西医院和包头中药有限责任公司书面许可，

不得私自使用、泄露、出版或以其他方式公开

目录

方案签名页㈠ 3
方案签名页㈡ 4
缩略语表 6
方案摘要 7
1 研究背景 9
1.1 概述 9
1.2 处方与功能主治 10
1.3 临床前药效学实验 10
1.4 临床前毒理学实验 10
1.5 既往临床研究 11
2 研究目的 11
3 临床试验依据 11
4 研究设计依据及研究方法 12
4.1 总体设计 12
4.2 随机化分组 12
4.3 盲法及盲法实施过程 12
4.4 对照药物的选择 13
4.5 试验场所和人员 13
5 研究对象的选择和退出 14
5.1 诊断标准 14
5.2 研究人群 15
6 样本量估算 17
7 治疗 18
7.1 研究药物 18
7.2 药物包装 18
7.3 药物的编码与标识 19
7.4 药物的分装与分配 19
7.5 用药方案 20
7.6 伴随用药 21
7.7 药物的清点与依从性评估 21
7.8 试验用药物的保存与回收 22
7.9 试验过程及访视 22
8 观测项目 23
8.1 一般资料 23
8.2 安全性观测 23
8.3 排除检测项目 23
8.4 有效性观察 23
8.5 观察记录方法 24
9 疗效和安全性评估 24
9.1 疗效评估 24
9.2 安全性评估 25
10 不良事件的记录与报告 25
10.1 不良事件定义 25
10.2 不良事件的预期性 26
10.3 不良事件严重程度判断标准 26

10.4 不良事件与试验药物关系的判断标准........ 26
10.5 不良事件的处理........ 27
11 应急信件的拆阅与处理........ 28
12 试验的质量控制与保证........ 28
12.1 实验室的质控措施........ 28
12.2 临床试验前培训........ 29
12.3 提高受试者依从性的措施........ 29
12.4 质量控制和质量保证系统........ 29
13 数据管理........ 29
14 统计分析........ 30
14.1 一般原则........ 30
14.2 统计分析人群........ 30
14.3 统计分析方法........ 31
15 伦理原则........ 33
15.1 伦理审查........ 33
15.2 受益与风险........ 33
15.3 招募受试者........ 33
15.4 知情同意的过程........ 33
15.5 受试者的医疗和保护........ 34
15.6 受试者隐私的保护........ 34
16 伦理学要求........ 34
17 资料保存........ 35
17.1 研究者保存的资料........ 35
17.2 申办者保存的资料........ 35
17.3 研究者和申办者共同保存的资料........ 35
18 各方承担职责与论文发表规定........ 35
19 试验结束后的随访和医疗措施........ 35
20 试验总结........ 36
20.1 病例报告表验收和原始病历保存。........ 36
20.2 揭盲人员与方法........ 36
20.3 总结与小结........ 36
21 临床试验预期进度和完成日期........ 36
21.1 试验开始时间........ 36
21.2 中期协调会........ 36
21.3 完成时间........ 37
21.4 试验资料收集、统计及总结时间........ 37
22 参考文献........ 37
附件一 清风感咳颗粒临床试验流程图........ 38
附件二 清风感咳颗粒临床研究受试者须知........ 39
附件三 药物性肝病处理参考预案（SOP）........ 42
附件四 药物性肾损害处理参考预案（SOP）........ 43
附件五 咳嗽特异性生活质量问卷（CQLQ）........ 44
附件六 用药包装标签........ 46
附件七 支气管激发试验操作规程........ 47

方案签名页㈠

清风感咳颗粒治疗感染后咳嗽（风邪恋肺证）II 期临床试验
方案签名页

（版本日期：2011 年 01 月 23 日）

研究者签名

我已熟悉本研究方案，确认方案中包括研究实施的必要内容，明确与本研究方案有关的研究者职责。我同意按照本研究方案，药物临床试验质量管理规范（GCP）和赫尔辛基宣言（2000 年在爱丁堡修订）以及其它有关治疗性药物的现行法律、法规进行本项临床研究并做出研究报告。

研究单位名称：	四川大学华西医院	研究者姓名（楷体）：	毛兵
研究者签名：		签名日期：	
研究单位名称：	辽宁中医药大学附属医院	研究者姓名（楷体）：	徐艳玲
研究者签名：		签名日期：	
研究单位名称：	南京市第一医院	研究者姓名（楷体）：	马俭
研究者签名：		签名日期：	
研究单位名称：	包头市中心医院	研究者姓名（楷体）：	闫少锋
研究者签名：		签名日期：	
研究单位名称：	第四军医大学唐都医院	研究者姓名（楷体）：	金发光
研究者签名：		签名日期：	

申办者签名

申办者名称：	包头中药有限责任公司	负责人姓名（楷体）：	贾金良
负责人签名：		签名日期：	

方案签名页㈡

清风感咳颗粒治疗感染后咳嗽（风邪恋肺证）Ⅱ期临床试验

方案中心签名页

1. 申办者

我将根据《药物临床试验质量管理规范（GCP)》规定，认真履行申办者职责。负责发起、申请、组织、资助和监查本项临床研究，特别对临床研究中发生与研究相关的损害或死亡的受试者承担治疗及相应的经济补偿，向研究者提供法律上的担保。

申办单位：包头中药有限责任公司

申办者项目负责人(签名)：________________ ______年____月____日

2. 主要研究者

我将根据 GCP 规定，认真履行研究者职责。

我已收到研究者手册，已知晓该研究药物的研究过程，并被告知将及时收到更新的研究者手册。

我已阅读过此方案，本项研究将根据赫尔辛基宣言和中国 GCP 规定的道德、伦理和科学原则进行。我同意按照本方案设计及规定开展此项临床研究。

我将负责作出与临床相关的医疗决定，保证受试者在研究期间出现不良反应时得到及时、适当的治疗，我知道正确报告严重不良事件的要求，我将根据要求记录和报告这些事件。

我保证将数据准确、完整、及时、合法地载入病例报告表。我将接受申办者派遣的监查员或稽查员及药品监督管理部门的稽查和视察，确保临床研究的质量。

我同意研究结果用于药品注册，公开发表。

我将在研究开始前提供一份履历，呈送伦理委员会、并可能呈送药政管理部门审查。

组长单位：四川大学华西医院

主要研究者（签名)：________________ _______年____月____日

机构有关负责人（签名)：____________ _______年____月____日

参加单位：辽宁中医药大学附属医院

主要研究者（签名)：________________ _______年____月____日

机构有关负责人（签名）：______________ ______ 年____月____日

参加单位：南京市第一医院

主要研究者（签名）：________________ ______ 年____月____日

机构有关负责人（签名）：______________ ______ 年____月____日

参加单位：包头市中心医院

主要研究者（签名）：________________ ______ 年____月____日

机构有关负责人（签名）：______________ ______ 年____月____日

参加单位：第四军医大学唐都医院

主要研究者（签名）：________________ ______ 年____月____日

机构有关负责人（签名）：______________ ______ 年____月____日

3. 监查员

我将根据 GCP 规定，认真履行监查员职责。

我已阅读过此方案，我将根据《药物临床试验质量管理规范》规定，认真履行监查者职责；保证临床试验中受试者权益受到保障，研究记录与报告的数据真实、准确、完整无误。保证研究遵循已批准的方案、药物临床试验质量管理规范和有关法规。

监查员(签名)：______________________ _______年____月____日

4. 生物统计学分析者

我将根据 GCP 规定，认真履行统计人员职责。

统计单位：北京岐黄药品临床研究中心

主要统计者(签名)：____________________ ______年____月____日

缩略语表

缩写	中文全称	英文全称
AE	Adverse event	不良事件
ALP	Alkaline Phosphatase	碱性磷酸酶
ALT	Alanine aminotransferase	丙氨酸氨基转移酶
AST	Aspartate aminotransferase	门冬氨酸氨基转移酶
BUN	Blood urea nitrogen	血尿素氮
Cr	Creatinine	肌酐
CMH	Cochran-Mantel-Haenszel	CMH 统计方法
CRF	Case Report Form	病历报告表
ECG	Electrocardiogram	心电图
FAS	Full Analysis set	全分析集
GCP	Good Clinical Practice	药物临床试验质量管理规范
ICF	Informed consent forms	知情同意书
ICH	International Conference on Harmonization	国际协调会
IEC	Independent Ethics Committee	独立伦理委员会
OTC	Over-the-Counter	非处方药
PPS	Per Protocol Set	符合方案集
γ-GT	γ-Glutamyl Transpeptidase	γ-谷氨酰转移酶
SAE	Serious Adverse Event	严重不良事件
SFDA	China’s State Food and Drug Administration	国家食品药品监督管理局
SOP	Standard Operating Procedure	标准操作规程
STB	Serum Total Bilirubin	血清总胆红素
SS	Safety Set	安全性分析集

方案摘要

研究题目：清风感咳颗粒Ⅱ期临床试验

研究目的：评价清风感咳颗粒治疗感染后咳嗽（风邪恋肺证）有效性和安全性，并对剂量与有效性、安全的关系进行探索，为III期确证性临床试验提供依据。

研究设计：多中心、随机、双盲、剂量平行对照设计。

研究人群：符合感染后咳嗽（风邪恋肺证）诊断标准者，年龄 18～65 岁，性别不限。

纳入标准：（1）符合感染后咳嗽的西医诊断；

（2）中医辨证为风邪恋肺证；

（3）日间咳嗽症状计分≥2 分和/或夜间咳嗽症状计分≥2 分；

（4）病程≥3 周，且≤8 周；

（5）年龄在 18～65 岁之间；

（6）受试者知情同意并签署知情同意书；

排除标准：（1）支气管激发试验阳性者；

（2）X 线胸片异常者；

（3）咳嗽变异性哮喘（CVA）、上气道综合征（UACS）、嗜酸粒细胞性支气管炎（EB）、食管反流性咳嗽（GERC）以及其他病因引起的咳嗽患者；

（4）服用血管紧张素转换酶抑制剂（ACEI）的患者；

（5）合并心脑血管、肺、肝、肾和造血系统严重原发性疾病，或影响其生存的严重疾病，如肿瘤或艾滋病；

（6）ALT、AST>1.5 倍正常参考值上限；血肌酐异常；尿蛋白＞＋；血 Ig 升高；血嗜酸性粒细胞增多；血白细胞＜3.0×10^9/L 或＞10.0×10^9/L；和/或中性粒细胞(N)分类>80%；

（7）近 1 个月内服用过与试验药物功能主治类似药物患者；

（8）妊娠或准备妊娠妇女，哺乳期妇女；

（9）过敏体质或对该药物已知成分过敏者；

（10）精神上或法律上的残疾患者；

（11）近 3 个月内参加其他药物临床试验者；

（12）研究者认为不适宜参加临床试验的患者。

治疗方案：大剂量组：清风感咳颗粒 2 袋（12g）/次，2 次/日，温水冲服；

小剂量组：清风感咳颗粒 1 袋（6g）/次+清风感咳颗粒模拟剂 1 袋（6g）/次，2 次/日，温水冲服；

零剂量组：清风感咳颗粒模拟剂 2 袋（12g）/次，2 次/日，温水冲服。

疗程：10 天。

有效性评价指标：主要疗效指标：咳嗽症状消失时间；

次要疗效指标：1、咳嗽症状缓解时间；

2、咳嗽改善程度（咳嗽评分及 VAS）；

2、中医疗效；

3、咳嗽特异性生活质量问卷（CQLQ）。

安全性评价指标：血常规、尿常规、心电图、肝功能（ALT、AST、STB、ALP、γ -GT）、肾功能（Bun、Cr）；

样本量：共 180 例；其中大剂量组 60 例，小剂量组 60 例，零剂量组 60 例。

清风感咳颗粒Ⅱ期临床试验方案

清风感咳颗粒治疗感染后咳嗽（风邪恋肺证）有效性和安全性的

随机、双盲、剂量平行对照、多中心Ⅱ期临床试验

1 研究背景

1.1 概述

咳嗽是机体的防御反射，有利于清除呼吸道分泌物和有害物因子，但长期、频繁剧烈的咳嗽对患者的工作、生活和社会活动则可造成严重的影响。据调查因咳嗽就诊者占呼吸专科门诊的80%以上，而感染后咳嗽（postinfectious cough）是咳嗽的常见病因之一。具有上呼吸道感染病史的患者，大约11%-25%会发生感染后咳嗽，在流行季节，感染后咳嗽发生率可高达25%-50%。现代医学尚缺乏治疗感染后咳嗽公认有效的治疗药物。目前国内外咳嗽指南推荐的第一代抗组胺H1受体拮抗剂及中枢性镇咳药仅是一种对症治疗，部分患者虽有一定疗效，但停药后咳嗽容易复发，还常伴有嗜睡、困倦等副作用。此类患者最易被临床医生所疏忽，很多患者长期被误诊为“慢性支气管炎”或“支气管炎”，大量使用抗菌药物治疗无效，或者因诊断不清而反复进行各种检查，不仅增加了患者痛苦，也加重了患者的经济负担。

祖国医学将感染后咳嗽归属于外感咳嗽的范畴,认为其病因以六淫为主,而风为六淫之首,故以风邪为先导,如是《诸病源候论·咳嗽候》所述,“风咳,欲语因咳,言不得竟是也”。 外感热病，经清热解表治疗后，热退，身痛等症亦缓解，卫表之邪得以大部分祛除。但经常有余邪未尽，尤其是风邪羁肺，导致肺宣降失常，则余咳嗽等症状。因此感染后咳嗽以外感风邪为主要病因，以“风邪束肺，肺失宣降”为主要病机，表现出咳嗽、痰粘而少、咳嗽遇风加重、咽痒、咽干等症状，当以宣肺祛风、止咳化痰之法治之。

清风感咳颗粒是在多年临床经验基础上总结出的用之于感染后咳嗽的临床有效方药，由早期经验方“宣肺止咳剂”经临床使用和科研筛选精简而成。清风感咳颗粒处方由麻黄、青风藤、百部、紫菀组成，功能主治为宣肺祛风、止咳化痰。用于风邪恋肺证，症见咳嗽、痰粘而少、咽痒、咽干、咳嗽常因吸入冷热空气或刺激性气味诱发加重、胸闷，感染后咳嗽见上述证候者。

根据国家食品药品监督管理局 2010L00279 号临床试验批件的要求，受包头中药有限责任公司和北京岐黄药品临床研究中心委托，由四川大学华西医院为负责单位，辽宁中医药大学附属医院、南京市第一医院、包头市中心医院、第四军医大学唐都医院为参加单位，对清风

感咳颗粒治疗感染后咳嗽（风邪恋肺证）有效性和安全性作出初步评价。

1.2 处方与功能主治

处方组成：麻黄、青风藤、百部、紫菀。
功能主治：宣肺祛风、止咳化痰。用于感染后咳嗽，症见咳嗽、咽痒、咳嗽遇风加重、咯痰、咽干、胸闷。

1.3 临床前药效学实验

清风感咳颗粒2.8g/kg(相当于临床治疗量的2倍)、1.4g/kg(相当于临床治疗量)、0.7g/kg（相当于临床治疗量的 1/2 倍）连续灌胃给药 5 天,可使二氧化硫和氨水刺激所致小鼠咳嗽潜伏期明显延长，咳嗽次数明显减少；使小鼠气管段酚红排泌量明显增加；抑制醋酸所导致的小鼠腹腔毛细血管通透性。清风感咳颗粒 1.6g/kg（相当于临床治疗量的 2 倍）、0.8g/kg（相当于临床治疗量）、0.4g/kg（相当于临床治疗量的 1/2 倍）连续灌胃给药 5 天，可使乙酰胆碱和组胺喷雾导致豚鼠出现喘息性抽搐的潜伏期明显延长；清风感咳颗粒 1.8g/kg（相当于临床治疗量的 2 倍）、0.9g/kg（相当于临床治疗量）、0.45g/kg（相当于临床治疗量的 1/2 倍）连续灌胃给药 5 天，还可抑制角叉菜胶所导致的大鼠足跖肿胀。免疫功能试验显示，清风感咳颗粒可明显抑制二硝基氯苯所致小鼠迟发型变态反应所表现的耳肿胀，明显抑制血清溶血素的形成，但对小鼠单核巨噬细胞吞噬功能抑制作用不明显；即清风感咳颗粒具有免疫抑制作用，主要是抑制特异性体液免疫和细胞免疫功能，对非特异性免疫功能抑制作用不明显。

综上述：清风感咳颗粒具有镇咳、祛痰、平喘、抗炎和免疫功能抑制作用。

1.4 临床前毒理学实验

1.4.1 急性毒性试验研究

用昆明种小鼠灌胃给药对清风感咳颗粒进行急性毒性试验。受药物浓度和给药体积的限制，对小鼠一次灌胃给药无法测出半数致死量。经最大给药量测定，小鼠每日口服的最大给药量为 75g / kg（相当于生药 341g /kg），为临床推荐成人每日用药量的 524 倍。

综上述：故清风感咳颗粒按临床推荐剂量服用，毒副作用小，是安全可靠的。

1.4.2 长期毒性试验研究

用 Wistar 大鼠进行长期毒性试验。清风感咳颗粒 8.58g / kg.d（相当于成人每日临床用量的 60 倍，相当于生药 73.6g)、4.29g / kg.d（相当于成人每日临床用量的 30 倍，相当于生药 36.8g)、2.15g / kg.d（相当于成人每日临床用量的 15 倍，相当于生药 18.4g）连续给药

13 周，无一例动物死亡；各组大鼠一般状态、饮食量、体重、血液学和血液生化学各项指标、主要脏器指数均未见异常；系统尸解和病理组织检查，给药各组大鼠受检器官与对照组也无明显区别。经过 2 周停药恢复后，各项指标与对照组相比无明显的差异。

综上述：清风感咳颗粒长期大剂量服用对动物的行为活动、生长发育、器官组织、肝肾功能、血液系统等均无毒性作用，故以临床推荐的剂量使用是较安全的。

1.5 既往临床研究

清风感咳颗粒经多年临床经验及科研成果的总结而成，在临床中用以此为基本方的经验方药治疗患者千余例，取得良好疗效，受到患者的好评和肯定。同时，于 2003-2007 年完成两项相关课题研究，并获国家专利一项、科研成果一项。

通过与西药（茶碱联合开瑞坦）对照的临床研究表明，清风感咳颗粒（原名祛风宣肺颗粒）治疗感染后咳嗽的愈显率为 75.50%，总有效率为 92.50%；西药组愈显率为 35.00%，总有效率为 70.00%，统计分析有显著差异，清风感咳颗粒治疗感染后咳嗽疗效显著，疗效明显优于西药组。给药治疗后，多数患者于服药后 2-3 天后咽痒咳嗽明显减轻，5 天后咳嗽咽痒症状基本消失。

2 研究目的

评价清风感咳颗粒治疗感染后咳嗽（风邪恋肺证）有效性和安全性，并对剂量与有效性、安全的关系进行探索，为Ⅲ期确证性临床试验提供依据。

3 临床试验依据

⑴国家食品药品监督管理局：《药物临床试验质量管理规范》2003 年 9 月；

⑵国家食品药品监督管理局：《药品注册管理办法》2007 年；

⑶《赫尔辛基宣言》2008 年；

⑷《中华人民共和国药品管理法》2001 年；

⑸郑筱萸,主编.中药新药临床研究指导原则(试行).第 1 版.北京:中国医药科技出版社, 2002 年 5 月第 1 版；

⑹汪秀琴,熊宁宁主编:临床试验机构伦理委员会操作规程,科学出版社,2007 年；

⑺ICHE8:临床研究的一般原则 2007；

⑻ICHE10：临床试验中的对照组选择 2007；

⑼供临床医师参阅的药理、毒理结论综述；

⑽清风感咳颗粒的处方组成、功能主治等资料。

4 研究设计依据及研究方法

4.1 总体设计

试验设计类型：本项试验采用多中心、随机、双盲、剂量平行对照临床研究。

表 1 四川大学华西医院负责与以下分中心协同完成

中心名称	中心编号
四川大学华西医院	01
辽宁中医药大学附属医院	02
南京市第一医院	03
包头市中心医院	04
第四军医大学唐都医院	05

4.2 随机化分组

4.2.1 随机化方法

采用区组随机化方法，为保证大剂量组、小剂量组与零剂量（安慰剂）组的病例数相等，采用 SAS 统计软件，给定种子数，产生随机数及相应的药物及病例分配方案。

4.2.2 随机方法的隐藏

随机号采用不透光信封密闭，由各试验中心集中管理。

4.3 盲法及盲法实施过程

采用双盲法。本临床研究因安慰剂的颜色、气味无法与试验药做到完全一致，故采用如下措施对研究者进行盲法，以避免来自研究者的偏倚。所有研究药物内外包装均一致，且无法观察到内容物；进入研究单位后由专人保管、发放、回收，专职人员发药，所有接触药物的成员不得参与病例观察和疗效评价，观察病例和评价疗效的研究者不得接触到药物。监查员可以接触内包装完好的药物，但不得接触内包装破损的药物；所有回收的药物内包装均应完好无损，否则须由药物保管人员就地销毁并记录。

4.4 对照药物的选择

对照药：根据《中药新药临床研究指导原则》关于临床试验设计的要求，由于未检索到与清风感咳颗粒功能主治相似的阳性对照药，加之现代医学尚缺乏治疗感染后咳嗽公认有效的治疗药物，并且感染后咳嗽为自限性，多能自行缓解，故选择安慰剂对照。

4.5 试验场所和人员

4.5.1 申办者

申办单位：包头中药有限责任公司

负责人：贾金良

地址：内蒙古包头市东河区南海路

联系电话：13848253331

4.5.2 临床试验组长单位

四川大学华西医院

中心项目负责人：张瑞明

联系电话：028-85423028

E-mail：zhruim-001@163.com

主要研究者:毛兵

联系电话：028-85423546

4.5.3 临床试验参加单位

⑴辽宁中医药大学附属医院

中心项目负责人：王文萍

联系电话：024-86291549

E-mail：Lnzyjd@sina.com

主要研究者:徐艳玲

联系电话：024- 86291600-1131

⑵南京市第一医院

中心项目负责人：孔小红

联系电话：025-86621776

E-mail：kfhnfh@sina.com

主要研究者:马俭

联系电话：18951670206

⑶包头市中心医院

中心项目负责人：刘清平

联系电话：0472-6955216

E-mail：qpliu202@sina.com

主要研究者:闫少锋

联系电话：0472-6955070

⑷第四军医大学唐都医院

中心项目负责人：刘琳娜

联系电话：029-84777631

E-mail：liulinna@fmmu.edu.cn

主要研究者:金发光

联系电话：029-84777425

5 研究对象的选择和退出

5.1 诊断标准

5.1.1 西医诊断标准

参照 [中华医学会呼吸病学分会哮喘学组. 咳嗽的诊断与治疗指南(2009 版). 中华结核和呼吸杂志. 2009. 32(6)：407-413]感染后咳嗽相关内容制定。

⑴咳嗽出现前有呼吸道感染病史；

⑵临床表现为刺激性干咳或咳少量白色粘液痰；

⑵通常持续 3～8 周；

⑷X 线胸片检查无异常；

⑸肺功能检查正常者；

⑹排除其它病因引起的咳嗽。

5.1.2 咳嗽症状计分标准

参照“咳嗽的诊断与治疗指南（2009 版）中华医学会呼吸病学分会哮喘学组”制定。

咳嗽症状积分共分为日间积分和夜间积分两部分，每部分均按照不同的轻重程度划分为 0—3 分 4 个等级。该评分体系反映了咳嗽频率、强度以及生活质量受影响的状况。

计分	日间咳嗽症状计分	夜间咳嗽症状计分

0 分	无咳嗽	无咳嗽
1 分	偶有短暂咳嗽	入睡时短暂咳嗽或偶有夜间咳嗽
2 分	频繁咳嗽，轻度影响日常生活	因咳嗽轻度影响夜间睡眠
3 分	频繁咳嗽，严重影响日常生活	因咳嗽严重影响夜间睡眠

5.1.4 中医辨证

风邪恋肺证

参考［国家中医药管理局发布，中医病证诊断疗效标准.南京大学出版社,1994 年，以及中药新药临床研究指导原则（试行），中国医药科技出版社，第 1 版，2002 年］咳嗽风邪恋肺证相关内容制定。

主症：咳嗽；

次症：①咽痒不适、咳嗽遇风加重；②咯痰黏少、咽干、胸闷；

舌象：舌尖红，苔白或黄；

脉象：脉平或浮。

以上主症必备，次症①必备,②具备 1 项或以上，结合舌脉，即可辨证为风邪恋肺证。

5.1.5 中医症状分级标准

表 2　主症分级计分标准

症　状	无	轻（2 分）	中（4 分）	重（6 分）
咳嗽	无	白天间断咳嗽，或入睡时短暂咳嗽，不影响工作生活；	白天咳嗽或见夜间咳嗽，尚能坚持工作；	昼夜频咳或阵发，影响工作和休息。

表 3　次症分级计分标准

症　状		无	轻（1 分）	中（2 分）	重（3 分）
咽痒不适		无	咽微痒	咽痒欲咳，咳后缓解	咽痒明显，咳后不缓解
咳嗽遇风加重		无（0 分）		有（2 分）	
咯痰黏少		无（0 分）		有（2 分）	
咽干		无	偶觉咽干	时觉咽干	明显，持续不解
胸闷		无	轻微胸憋	胸闷明显，时有太息	胸闷如窒
舌脉象	舌质	舌尖红　☐			其它：
	舌苔	白　☐		黄　☐	其它：
	脉象	平　☐		浮　☐	其它：

5.2 研究人群

5.2.1 纳入标准

⑴符合感染后咳嗽的西医诊断；

⑵中医辨证为风邪恋肺证；

⑶日间咳嗽症状计分≥2 分和/或夜间咳嗽症状计分≥2 分；

⑷病程≥3 周，且≤8 周；

⑸年龄在 18～65 岁之间；

⑹受试者知情同意并签署知情同意书。

5.2.2 排除标准

⑴支气管激发试验阳性者；

⑵X 线胸片异常者；

⑶咳嗽变异性哮喘（CVA）、上气道综合征（UACS）、嗜酸粒细胞性支气管炎（EB）、食管反流性咳嗽（GERC）以及其他病因引起的咳嗽患者；

⑷服用血管紧张素转换酶抑制剂（ACEI）的患者；

⑸合并心脑血管、肺、肝、肾和造血系统严重原发性疾病，或影响其生存的严重疾病，如肿瘤或艾滋病；

⑹ALT、AST>1.5 倍正常参考值上限；血肌酐异常；尿蛋白＞＋；血 Ig 升高；血嗜酸性粒细胞增多；血白细胞＜3.0×109/L 或＞10.0×109/L；和/或中性粒细胞(N)分类>80%；

(7)近 1 个月内服用过与试验药物功能主治类似药物患者；

(8)妊娠或准备妊娠妇女，哺乳期妇女；

⑼过敏体质或对该药物已知成分过敏者；

(10)精神上或法律上的残疾患者；

(11)近 3 个月内参加其他药物临床试验者；

(12)研究者认为不适宜参加临床试验的患者。

研究者认为不适宜参加临床试验的患者。凡符合上述排除标准一项或一项以上者，均不能纳入临床试验。

5.2.3 剔除标准

⑴纳入后发现不符合纳入标准者；

⑵未按试验要求服用研究药物（试验药物或安慰剂），依从性＜80% 或＞120%者；

⑶不能合作者，包括不按计划完成随访等。

5.2.4 退出标准

⑴出现病情加重，如服药 24 小时后合并明显喘息症状等，根据医生判可退出临床试验。受试者按常规处理，该病例按无效处理，最后一次数据应转接进入全分析集（FAS）；

⑵出现过敏反应或严重不良事件者，根据医生判断可退出临床试验；

⑶受试者在临床试验过程中不愿意继续进行临床试验，向主管医生提出退出临床试验的要求，可以退出临床试验；

⑷受试者依从性差，未按规定用药，违背试验方案，根据医生判断可退出临床试验。

5.2.5 中止及终止临床试验的标准

⑴服药过程中，出现严重不良事件；

⑵试验中发现治疗药物效果不好，甚至无效，不具有临床价值，作为无效中止试验；

⑶试验中发现临床试验方案有重大失误，难以评价药物效应；或设计较好的方案在实施中发生了重大偏差（如盲底泄露等）；

⑷药品监督管理部门因某种原因勒令终止试验；

⑸申办者要求终止（如经费原因、管理原因等）。

5.2.6 病例的脱落及处理

⑴脱落的定义

所有填写了知情同意书并筛选合格进入试验的患者，均有权利随时退出临床试验，无论何时何因退出，只要没有完成方案所规定观察周期的受试者，称为脱落病例，包括以下几种情况：

- 受试者出现严重不良事件，根据医生判断该病例应该停止临床试验；

试验过程中受试者原有病情加重，或试验中出现了其它影响试验观察的病征，根据医生判断该病例应该停止临床试验，并作无效病例处理；

- 临床试验方案实施中发生了重要偏差，如依从性太差等，难以评价药物效应；
- 受试者不愿意继续进行临床试验，向主管医生提出退出临床试验或虽未提出退出试验，但已失访。

⑵脱落病例的处理

对脱落的受试者，研究者应采取登门、预约随访、电话、信件等方式，尽可能与受试者联系，询问理由、记录最后一次服药时间、完成所能完成的评估项目。对于因过敏或其他不良反应、治疗无效而退出试验的病例，研究者应根据受试者的实际情况，采取相应的治疗措施。研究者应妥善保存脱落病例的有关试验资料，既作留档，也是进行全分析集统计所需。

6 样本量估算

⑴本项目临床试验的大剂量组、小剂量组和零剂量（安慰剂）组，按 1∶1∶1 的比例分配；

⑵根据《药品注册管理办法》附件一“中药、天然药物注册分类及申报资料要求”中的规定，

临床试验的最低病例数（试验组）要求：Ⅱ期为 100 例。本试验为剂量探索，并考虑到试验中可能的脱落/剔除，最终总的样本量估算为180例，其中大剂量组60例、小剂量组60例、安慰剂组（零剂量组）60例。

7 治疗

7.1 研究药物

7.1.1 药品来源

- 清风感咳颗粒，规格：6g/袋；生产批号：100901；储藏条件：密封、防潮；由包头中药有限责任公司提供；
- 清风感咳颗粒模拟剂，规格：6g/袋；生产批号：100902；储藏条件：密封、防潮；由包头中药有限责任公司提供。

7.2 药物包装

7.2.1 统一标签格式

按照盲法试验要求，3组用药在外观上保持一致，使用统一标签。包装标签式样见附件5。

7.2.2 药品包装

包装	用药时间	每盒/袋包装药品名称及数量		
		大剂量组	小剂量组	零剂量（安慰剂）组
第1小盒	第1～3天	试验药物6包，其中：清风感咳颗粒2袋/包<br>共计清风感咳颗粒12袋	试验药物6包，其中：清风感咳颗粒 1 袋 + 清风感咳颗粒模拟剂1袋/包<br>共计清风感咳颗粒6袋、清风感咳颗粒模拟剂6袋	试验药物6包，其中：清风感咳颗粒模拟剂2袋/包<br>共计清风感咳颗粒模拟剂12袋
第2小盒	第4～6天	试验药物6包，其中：清风感咳颗粒2袋/包<br>共计清风感咳颗粒12袋	试验药物6包，其中：清风感咳颗粒 1 袋 + 清风感咳颗粒模拟剂1袋/包<br>共计清风感咳颗粒6袋、清风感咳颗粒模拟剂6袋	试验药物6包，其中：清风感咳颗粒模拟剂2袋/包<br>共计清风感咳颗粒模拟剂12袋
第3小盒	第7～9天	试验药物6包，其中：清风感咳颗粒2袋/包<br>共计清风感咳颗粒12袋	试验药物6包，其中：清风感咳颗粒 1 袋 + 清风感咳颗粒模拟剂1袋/包<br>共计清风感咳颗粒6袋、清风感咳颗粒模拟剂6袋	试验药物6包，其中：清风感咳颗粒模拟剂2袋/包<br>共计清风感咳颗粒模拟剂12袋

第 4 小盒	第 10 天<br>+2 天	试验药物 6 包，其中：<br>清风感咳颗粒 2 袋/包<br>共计清风感咳颗粒 12 袋	试验药物 6 包，其中：<br>清风感咳颗粒 1 袋 + 清风感咳颗粒模拟剂 1 袋/包<br>共计清风感咳颗粒 6 袋、清风感咳颗粒模拟剂 6 袋	试验药物 6 包，其中：<br>清风感咳颗粒模拟剂 2 袋/包<br>共计清风感咳颗粒模拟剂 12 袋

7.3 药物的编码与标识

7.3.1 处理编码的产生

采用分层、区组随机化方法。随机数字表由统计专业人员提供。分别按中心进行分层，选取适合段长，借助 SAS 统计软件，给定种子数，产生 180 例受试者所接受处理的随机编码表。

7.3.2 应急信件的准备

⑴采用密封不透光的信封，信封表面印有该份药物编号和遇紧急情况揭盲的规定。如果拆阅，需注明拆阅者、拆阅日期、原因等，并在病例报告表中记录；

⑵信封内印有该受试者的服药信息、处理方法及应立即汇报的单位和地址。应急信件准备完成后，随药物发往各个中心，在试验结束后统一收回。

7.3.3 按处理编码对药物进行包装和编号

由与本次临床试验的临床观察、监查、统计分析等无关的人员，根据已形成的处理编码将相应的药物编号贴在试验用药物外包装盒醒目位置。

7.3.4 处理编码和药物分装的编盲记录

全部试验用药物编码过程由编盲者书写成文件形式，即编盲记录，作为该临床试验的文件之一保存。其内容包括：申办者药品的准备、药品的包装、用法、储存要求、药品发放办法、随机处理编码的产生、按每个受试者包装的药盒、应急信件、试验药与对照药的药检报告、盲底的保存、揭盲的规定和各个中心分配药盒的编号等。

7.3.5 包装后药物的分发

将分装好的试验用药盒按随机分层的中心编号，与相应药物编号的应急信件发往各个试验中心。

7.3.6 盲底保存

全部处理编码所形成的盲底连同产生随机数的随机种子、区段的长度等参数，密封后一式两份分别交四川大学华西医院和申办者两处妥善保存，试验期间盲底不得拆阅。如果发生了任何非规定情况所致的盲底泄露，并影响了该试验结果的客观性，则该试验将被视为无效。

7.4 药物的分装与分配

7.4.1 随机方法

本试验方案采用分层、区组随机化方法。采用 SAS 6.12 Proc PLAN 程序步，给定种子数，产生随机数，即列出流水号为 001～180 所对应的随机化治疗分配方案。受试者按照将按 1∶1∶1 比率随机进入大剂量组、小剂量组、零剂量组。按随机确定的各医院分配的药物编号，各中心筛选病人，合格者入选，由药品管理员按每位受试者入组时间先后顺序和药物编号依次发放药物，不得选择药物。申办单位将提供给每位患者足够全疗程的同一药物编号的研究药物。发药时，药品管理员应及时填写“试验药品发放记录表”。每一编号的试验药物均附有一份相应编号的应急信件，应急信件保存在各试验单位的主要研究者处。试验单位及病例分配见表 4。

表 4 临床试验单位及病例分配表

临床试验中心	编号	病例数	大剂量组	小剂量组	零剂量组	药物编号
四川大学华西医院	01	36	12	12	12	001～036
辽宁中医药大学附属医院	02	36	12	12	12	037～072
南京市第一医院	03	36	12	12	12	073～118
包头市中心医院	04	36	12	12	12	119～144
第四军医大学唐都医院	05	36	12	12	12	145～180

注：各中心脱落或剔除应不超过 20%

7.4.2 受试者分配

按照各中心分配的中心随机编号，依照时间先后顺序纳入受试者，依照从小到大的顺序，分配所对应号的治疗药物进行了治疗。

7.4.3 研究药物发放和受试者培训

医生应按每位患者就诊先后顺序依药物编号从小到大顺序发放药物，不得选择药物，该药物编号将在整个试验过程中保持不变。发药时，药物管理人员应及时、准确填写药物发放记录表。药物管理人员对受试者进行相应的指导，按照正确的要求服用药物。

临床试验开始前应对参加临床试验的人员进行统一培训，使研究者对于临床试验方案的理解和认识基本一致。

7.5 用药方案

7.5.1 用药方法

大剂量组：清风感咳颗粒 2 袋（12g）/次，2 次/日，温水冲服；

小剂量组：清风感咳颗粒 1 袋（6g）/次 + 清风感咳颗粒模拟剂 1 袋（6g）/次，2 次/日，温水冲服；

安慰剂组：清风感咳颗粒模拟剂 2 袋（12g）/次，2 次/日，温水冲服。

7.5.2 疗程

连续服药 10 天

7.6 伴随用药

伴随用药是指受试者除研究药物以外使用的其它药物（包括急救药物），分为允许用药和禁用药。

7.6.1 允许使用的伴随药物

⑴合并其它疾病必须继续用药的其它药物和治疗方法是允许的，必须在合并用药表中详细记录药名（或其它疗法名）、用量、使用次数和时间等，以便总结时加以分析和报告；

⑵纳入后的受试者均应嘱注意休息、保持口腔清洁、戒烟、注意营养、饮食要易于消化，密切观察并防治并发症。

7.6.2 禁用的伴随药物

⑴不得使用抗菌类药物；

⑵不得使用对咳嗽有治疗作用或与试验药物功能主治相似的中药汤剂、中成药、西药；一旦使用禁用药物，则受试者退出研究。

7.7 药物的清点与依从性评估

7.7.1 药物清点

在受试者每次复诊时，观察医师如实记录受试者接受、服用和归还的药品数量，判断受试者服药依从性，并决定该患者是否能继续参加试验，同时及时记录在研究病例和病例报告表中。

7.7.2 依从性评估标准

受试者治疗的依从性根据受试者日志记录的实际用药量和试验计划用药量来计算，计算公式如下：

$$\frac{\text{实际使用总剂量}}{\text{计划使用总剂量}} \times 100\% = \text{依从性}$$

实际使用总剂量为受试者每日用药剂量之和（袋数），计划使用总剂量根据受试者计划

用药天数和研究疗程（7天）计划日剂量来计算。

如果受试者依从性<80%或>120%，则视为主要方案违背。

7.8 试验用药物的保存与回收

7.8.1 药物保存

试验用药物由试验单位统一保存、专人管理（温度15～25℃），并有相应的保存和管理程序。发放于患者的药物需在干燥、阴凉处保存；

7.8.2 药物回收

回收药物交于药品管理员，试验完成后，回收药物及未用药物由申办单位统一回收、统一销毁。

7.9 试验过程及访视

7.9.1 基线访视（第0天）

最初的筛选评估将在试验开始时进行。在筛选工作之前，向受试者说明试验目的、方法、利益与风险等，以及签署书面的知情同意书。其工作步骤如下：

- 受试者自愿签署书面知情同意书；
- 取得病史及人口学资料；
- 进行全面的问诊及体格检查，包括：症状、肺部听诊、体温、舌象、脉象等；
- 患者填写咳嗽特异性生活质量问卷（CQLQ）量表；
- 实验室检查：尿妊娠实验（育龄期妇女）、胸片、支气管激发或舒张试验；
- 进行实验室安全性检查，包括血、尿、肝、肾功能、心电图等；
- 按入选顺序发放试验用药，并在 CRF 中登记相应的药物编号，且该药物编号在整个研究中保持不变；填写药品发放登记表；
- 告知下次随访时间。

7.9.2 访视 1（第 6±1 天）

- 回收药品，评价服药依从性，不合格者剔除；
- 进行全面的问诊及体格检查，包括：症状、肺部听诊、体温、舌象、脉象等；
- 发药品并填写药品发放登记表；
- 记录不良事件和纠正治疗；

◆ 告知下次随访时间。

7.9.3 访视 2（第 11±2 天）

◆ 回收药品，包括包装材料，评价服药依从性；

◆ 进行全面的问诊及体格检查，包括：症状、肺部听诊、体温、舌象、脉象等；

◆ 记录不良事件；

◆ 进行安全性实验室检查：肝肾功能、血尿常规和心电图等；

◆ 患者填写咳嗽特异性生活质量问卷（CQLQ）量表。

以上参见清风感咳颗粒临床试验流程图

8 观测项目

8.1 一般资料

包括人口学、家族史、既往史、合并疾病及治疗史、现用药史与试验研究相关的症状和体征等。

8.2 安全性观测

⑴一般体检项目，包括肺部听诊、体温、呼吸、心率、血压、身高、体重等；

⑵血、尿常规检查；

⑶肝功能（ALT、AST、STB、ALP、γ-GT）、肾功能（Bun、Cr）检查；

⑷心电图检查；

以上项目于治疗前后各检查一次。

8.3 排除检测项目

⑴胸片（正位）；

⑵支气管激发或舒张试验；

⑶尿妊娠实验（育龄期妇女）。

以上项目于筛选期检查一次。

8.4 有效性观察

⑴咳嗽症状消失时间；

⑵咳嗽症状缓解时间；

⑶咳嗽改善程度（咳嗽评分及 VAS）；

⑷中医疗效；

⑸咳嗽特异性生活质量问卷（CQLQ）。

8.5 观察记录方法

观察期间，按要求的相应时间认真填写研究病例与病例报告表，症状按症状分级标准分别记分，无症状或症状消失，记为0分；舌脉不记分；其余项目用文字填写。

9 疗效和安全性评估

9.1 疗效评估

9.1.1 主要疗效指标

主要疗效指标：咳嗽（西医）消失时间。

9.1.2 次要疗效指标

次要疗效指标：⑵咳嗽症状缓解时间；

⑶咳嗽改善程度（咳嗽评分及 VAS）；

⑷中医疗效；

⑸咳嗽特异性生活质量问卷（CQLQ）。

9.1.3 疗效评定标准

⑴咳嗽缓解时间判定标准

患者达到日间且夜间咳嗽症状计分≤1分或下降1个等级，并持续48小时者，称为“缓解”。

记录观察期内患者达到咳嗽缓解的时间。

若患者日记卡的咳嗽症状计分连续两天均为日间且夜间咳嗽症状计分≤1分（注：咳嗽症状计分评估的是患者前24小时的咳嗽情况），则“咳嗽缓解时间”记为后一天。例如第5天和第6天患者日记卡上的日间且夜间咳嗽症状计分均≤1分，则咳嗽缓解时间记为“6天”。

对咳嗽缓解时间进行三组治疗前后比较。

⑵咳嗽疗效判定标准

对咳嗽症状计分、咳嗽程度进行三组治疗前后比较，并统计症状的改善程度以及消失率。

⑶中医证侯疗效判定标准

1、痊愈：中医证候临床症状基本消失，疗效指数为≥95%；

2、显效：中医证候临床症状明显改善，70%≤疗效指数＜95%；

3、有效：中医证候临床症状改善，30%≤疗效指数＜70%；

4、无效：中医证候临床症状无改善，疗效指数＜30%。

$$\text{中医证侯疗效指数} = \frac{\text{治疗前中医症状积分}-\text{治疗后中医症状积分}}{\text{治疗前中医症状积分}} \times 100\%$$

⑷咳嗽特异性生活质量问卷（CQLQ）

比较各组患者治疗前后咳嗽特异性生活质量问卷（CQLQ）积分变化，并进行组间变化的差值比较。

9.2 安全性评估

9.2.1 试验前后生命体征的变化

血压、呼吸、心率、心律。

9.2.2 实验室检查的各项标准

血、尿常规，肝功能（ALT、AST、STB、ALP、γ-GT）、肾功能（Bun、Cr）检验以及心电图（正常标准参考本医院实验室检查正常范围）

9.2.3 不良事件的发生率

9.2.4 严重不良事件的发生率

10 不良事件的记录与报告

10.1 不良事件定义

不良事件是指在临床研究观察期间出现的影响患者健康的任何症状、综合征或疾病，也包括了实验室或其他诊断过程中发现的与临床相关的情况，如需要计划外诊治措施，或导致从研究中退出，或有临床意义的实验室检查项目异常等。

不良事件可能是：新的疾病；治疗状态症状或体征的恶化，或伴随疾病的恶化；对照药物的作用；与参加该试验无关；一个或多个因素的组合。所以，“不良事件”这一术语并不意味着与试验药物有因果关系。

严重不良事件是指研究药物在任何剂量下或在观察期间任何时候出现的以下不良事件，包括：导致死亡；即刻危及生命；需住院治疗或延长住院时间；导致永久的或严重的残疾；超剂量而引起的；引起癌症；导致先天畸形；有重要的医学意义（指那些不会立即危及生命

或导致死亡或需住院的事件，但可能危害患者或需要采取措施来预防上述所定义的一种后果）；需要医学处理来防止永久性的损伤或损害。

10.2 不良事件的预期性

- 预期：其性质或严重性与适用的产品信息（研究者手册）一致；
- 非预期：其性质或严重性与适用的产品信息（研究者手册）不一致；

10.3 不良事件严重程度判断标准

- 轻度：轻度不适，受试者可以忍受，不影响继续用药，不需作特殊处理；
- 中度：中度不适，需作对症处理，能够坚持用药；
- 重度：重度不适，患者不能耐受，需停止试验。

10.4 不良事件与试验药物关系的判断标准

不良事件与受试药的关系应分为五级，即：肯定有关，很可能有关，可能有关，可能无关，肯定无关。具体判断标准如下：

肯定有关：某临床事件包括实验室检查异常，其发生与研究用药有合理的时序关系，且不能用伴随疾病或其它药物来解释。停药反应有临床合理性。该事件从药理学或临床表现方面须与研究药物有肯定相关性，如果需要，用再给药来确认；

很可能有关：某临床事件包括实验室检查异常，与研究用药有合理的时间顺序，不太可能是由于伴随疾病或其它药物引起。停药反应有临床合理性。不需要再次给药来确认；

可能有关：某临床事件包括实验室检查异常，与研究用药有合理的时间顺序，但也可以由伴随疾病或其它药物来解释。停药信息可以缺失或不清；

可能无关：某临床事件包括实验室检查异常，与研究用药有时序关系，但与研究药物可能无因果关系，其它药物或疾病可对因果关系提供合理的解释；

无关：不满足以上任何一条的不良事件；

	A	B	C	D	E
肯定有关	+	+	−	+	+
很可能有关	+	+	−	+	?

可能有关	+	+	±	±	?
可能无关	+	−	±	±	?
无关	−	−	+	−	−

注：+ 表示肯定，- 表示否定，± 表示肯定或否定，？ 表示情况不明。

满足下列分类之一的不良事件被认为是不良药物反应：肯定有关、很可能有关、可能有关。

研究者不能将不良事件归类为无法判断，在证据不充分、相互矛盾等情况下应随访以作进一步澄清。

10.5 不良事件的处理

10.5.1 观察与记录

研究者应要求患者如实反映用药后的病情变化，避免诱导性提问。试验期间出现的任何不良反应均应填写“不良事件表”，并追踪调查，详细记录处理经过及结果，直到化验检查恢复正常，症状体征消失。追踪随访方式可以根据不良反应的轻重选择住院、门诊、家访、电话、通讯等多种形式。

10.5.2 医疗处理

发现不良反应时，研究者应根据病情决定诊治措施，并决定是否中止观察。出现严重不良事件，承担临床研究的单位须立即采取必要处理措施，保护受试者安全。

10.5.3 严重不良事件报告制度

研究者应填写《严重不良事件报告表》，在 24 小时内分别报告国家食品药品监督管理局药品注册司、安全监管司和省级药品监督管理局、申办者及伦理委员会，并在报告上签名及注明日期。申办者还应及时向各参加单位通报，并保证满足所有法律法规要求的报告程序。

紧急情况，包括严重，特别是致死的不良反应，应以最快的通讯方式（包括电话、传真、特快专递、E-mail 等）向所在省、自治区、直辖市药品不良反应监测专业机构报告

表 5 严重不良事件报告各单位联系人及其电话和传真

单位	联系人	联系电话	传真
包头中药有限责任公司	贾金良	0472-4606435	0472-4606435
北京岐黄药品临床研究中心	林　丹	010-88820558	010-88820550
四川大学华西医院医院伦理委员会	孙荣国	18980602110	

国家食品药品监督管理局药品注册司研究监督处		010-88331033	010-88331023
四川大学华西医院	张瑞明	028-85423028	028-85423028

10.5.4 未缓解的不良事件的随访

所有在疗程结束时尚未完全缓解的不良事件(包括药品不良反应)，均应追踪观察至妥善解决或病情稳定。

10.5.5 关于临床实验室检查异常

某些异常的实验室检查结果(如临床生化、血液学、尿液分析)经研究者判定具有临床意义，如果它们符合不良事件的定义，或严重不良事件定义，则必须将其作为不良事件或严重不良事件予以记录。在服药后发现的，或者在基线评估时存在并在研究开始后加重的有临床意义的异常实验室检查结果及其它异常所见，也应作为不良事件或严重不良事件。但是，与所研究疾病有关的有临床意义的异常实验室检查结果或其它异常所见，除非研究者判定较所预料的受试者的病情更为严重，否则不包括在不良事件或严重不良事件中。在研究开始时存在或被发现但未加重的异常实验室检查结果或其它异常所见，也不包括在不良事件和严重不良事件中。但是如果研究者认为针对实验室检查异常的程度应该给予必要的治疗时，这种实验室检查应作为不良事件记录，同时要记录伴随治疗。所有异常的实验室检查都应随访至缓解或稳定为止。

11 应急信件的拆阅与处理

当患者发生紧急情况，需立即查明某一患者服药的种类时，研究单位的主要研究者可将随每份药品下发的相应编号的应急信件拆阅，内有该患者所服药物编号的药品名称及常见不良反应处理方法。同时立即将处理结果通知临床监查员。研究人员应在病例报告表上详细记录揭盲的理由、日期，并签字，同时填写不良事件报告表，逐级上报医院伦理委员会及省市药品监督管理局。自试验结束时不良事件仍未缓解者，根据具体情况随访至病情痊愈或稳定。

注意一般情况下，研究者不得轻易揭盲。如有揭盲，则该患者将被中止试验。

12 试验的质量控制与保证

12.1 实验室的质控措施

实验室要建立实验观测指标的标准操作规程和质量控制程序。实验室检测结果必须用计算机打印。数据应具有可溯源性。

12.2 临床试验前培训

申办者负责组织专家在临床试验开始前对研究者进行试验方案的培训，对所有量化标准进行一致性检验。签署研究者声明。

12.3 提高受试者依从性的措施

研究者应认真执行知情同意，使受试者充分理解试验要求，配合试验。
采用药物计数法，监测受试者服药依从性。依从性＝（实际服用量 / 应该服用量）×100%。依从性＜80%或＞120%视作对试验方案的重大违反。

12.4 质量控制和质量保证系统

申办者及研究者均应履行各自职责，并严格遵循临床试验方案，采用标准操作规程，以保证临床试验的质量控制和质量保证系统的实施。

本研究整个过程中将遵照 GCP 原则，研究者应如实、详细、认真记录 CRF 中各项内容。对临床试验中所有观察结果和发现都应加以核实，保证数据的可靠性。实验室各项检测应严格遵循 SOP。北京岐黄药品临床研究中心将指派临床监查员定期对研究医院进行现场监查访问，以保证研究方案中所有内容都得到严格遵守和研究资料填写正确。

监查员应具有医学背景。申办者任命监查员，保障参加临床试验受试者的权益，保证试验数据记录与报告真实、准确、完整无误，保证试验遵循已批准的方案、《药品临床试验管理规范》和有关法规。监查员访视的次数要能满足临床试验质量控制的需要。监查员每次访视后应向机构项目负责人通报监查结果。

13 数据管理

数据管理详见“数据管理计划书”，在数据库锁定前，根据数据特征，数据管理人员将与主要研究者、申办者一同进行盲态核查并进行讨论，并最终形成数据管理报告。本方案仅提供数据管理的一般要求。

13.1 病例报告表(CRF)的填写与移交：完成的病例报告表由临床研究者和监察员审查后，交数据统计单位，进行数据录入与管理工作。所有过程均需记录。

13.2 数据的录入与修改：数据录入与管理由统计单位数据管理员负责。采用EpiData3.1数据库，进行数据录入与管理。为保证数据的准确性，应由两个数据管理员独立进行双份录入并校对。

对病例报告表中存在的疑问。数据管理员将填写疑问解答表(DRO)，并通过临床监查员向

研究者发出询问，研究者应尽快解答并返回，数据管理员根据研究者的回答进行数据修改，确认与录入，必要时可以再次发出DRO。

13.3 盲态核查与数据库锁定：在盲态审核并确认所建的数据库无误后，由主要研究者、申办者、统计分析人员对数据库进行锁定。

13.4 揭盲：研究数据全部核查完毕并锁定后，由主要研究者（PI）、统计人员和申办方共同讨论统计计划书，并揭盲，三方人员在盲底签字。

揭盲后，对数据库的任何修改，需由临床研究负责人、生物统计学家和数据管理员共同达成书面同意方可进行。

盲底交由统计分析人员按统计计划书进行统计分析，最后由统计分析人员写出统计分析报告，主要研究者根据统计报告写出临床试验总结报告。

14 统计分析

14.1 一般原则

所有的统计检验均采用双侧检验，P值小于或等于0.05将被认为所检验的差别有统计意义（特别说明的除外）。当多组之间的差别有统计学意义时，进一步两两比较，P值小于或等于0.0167被认为所检验的差别有统计意义。

定量指标的描述将计算例数、均数、标准差、中位数、最小值、最大值。分类指标的描述用各类的例数及百分数。

14.2 统计分析人群

全分析集(Full Analysis Set，FAS)：指尽可能接近意向性分析原则的理想的受试者集(包括所有随机化入组，并至少接受一次治疗的全部受试者)。该数据集由所有随机化入组的受试者以最小的和合理的方法剔除构成，如对入组后无任何随访观察数据的病例(无任何用药)可以剔除；对主要变量缺失值的估计，如未能观察到全部治疗过程的病例资料，采用最接近一次观察的结果结转(carry-forward)到试验数据缺失处，各组在终点时评价疗效的受试者数与试验开始于保持一致。

符合方案集(Per Protocol Set，PPS)：所有符合试验治疗方案、依从性好、使用试验药物数量在80%-120%、完成CRF规定填写内容的病例，主要变量可以测定，基线变量没有缺失，没有对试验方案的重大违反。

安全集(Safety Analysis Set，SS)：所有随机化后至少接受一次治疗的受试者。

本试验中，基线资料的分析采用FAS分析。主要疗效指标同时对FAS和PPS进行分析。但以FAS的结论为主。当FAS和PPS所得结论一致时，可以增加结论的可信度。安全性评价采用SS分析。

14.3 统计分析方法

14.3.1 入选及完成情况

列出各个中心入组及完成病例情况及脱落原因，描述入组病例及安全性、有效性分析数据集的例数。列出未进入PPS者清单。

14.3.2 基线均衡性分析

基线定义为受试者入组（服药前）。

基础值的均衡性分析针对病例入组时基本人口学特征、生命体征、疗效相关指标等，以说明三组基础情况是否可比。其中，定量指标(如三组的年龄、体温、心率、呼吸、收缩压、舒张压、中医证候积分等)列出例数、均数、标准差、中位数、最大值、最小值，采用F检验/秩和检验对三组进行比较；定性指标(如性别、婚姻状况、心律、过敏史、治疗史等)，列出频数及百分数，并采用χ^2检验/Fisher精确概率法进行比较。

14.3.3 影响试验评价因素比较

合并用药情况分析

列出频数及百分数，并采用卡方检验／Fisher精确检验进行比较。

用药依从性分析

列出用药＜80%或＞120%、80%～120%的频数及百分数，并采用χ^2检验／Fisher精确检验进行比较。

暴露于治疗的时间

治疗时间（天）=末次用药时间－首次用药时间＋1

计算例数、均数、标准差、中位数、最小值、最大值，采用F检验/秩和检验对三组进行比较。

14.3.4 有效性分析

⑴咳嗽缓解及消失时间

采用Log-rank检验进行生存分析比较。

⑵咳嗽疗效

按照疗效判定标准计算各类的例数和百分比，采用按中心分层的CMH-χ^2检验对三组进行比较。

按照愈显率和总有效率分别计算各类的例数和百分比，采用按中心分层的CMH-χ^2检验对三组进行比较，并分别计算三组率差的95％可信区间。

⑶咳嗽症状、程度积分的变化情况

描述各访视点等较基线的变化情况，计算例数、均数、标准差、最小值、最大值、中位数，采用协方差分析（ANCOVA）模型对组间治疗前后的变化差值进行比较，此分析模型以基线数据为协变量，考虑分组、中心的作用。并以此模型为基础，分别计算各组治疗前后积分差值及三组差值之差的最小二乘均数（LSMEAN）及95%可信区间。此外，为考察各中心的一致性，还将在上述协方差分析模型的基础上，增加一个含有中心与分组交互项的协方差分析模型，当P＜0.10时，判断为中心与分组间存在交互作用。对各治疗组治疗前后的变化采用配对t检验进行比较。

⑷中医证候疗效

按照痊愈、显效、有效、无效计算各类的例数和百分比，采用按中心分层的CMH-χ^2检验对三组进行比较。

按照愈显率和总有效率分别计算各类的例数和百分比，采用按中心分层的CMH-χ^2检验对三组进行比较，并分别计算三组率差的95％可信区间。

⑸中医证候积分的变化情况

描述各访视点等较基线的变化情况，计算例数、均数、标准差、最小值、最大值、中位数，采用协方差分析（ANCOVA）模型对组间治疗前后的变化差值进行比较，此分析模型以基线数据为协变量，考虑分组、中心的作用。并以此模型为基础，分别计算各组治疗前后积分差值及三组差值之差的最小二乘均数（LSMEAN）及95%可信区间。对各治疗组治疗前后的变化采用配对t检验进行比较。

14.3.5 安全性分析

⑴与安全性有关的实验室检查

对实验室检查以治疗前后交叉表(根据临床判断)的形式列出所有完成的检查项目，并列出疗后异常的检查项目。

⑵不良事件

试验期间所有不良事件的种类、严重程度及与试验药物的关系等将列表描述。

不良事件将按全部不良事件、相关不良事件分类列表给出，其中，出于保守的考虑，相关不良事件将包括与研究药物“肯定有关”、“很可能有关”、“可能有关”、“可疑有关”的不良事件。

⑶生命体征

描述体征(体温、心律、呼吸、收缩压、舒张压)治疗前后的变化，计算例数、均数、标准差、中位数、最小值、最大值，采用方差分析/秩和检验进行比较。

15 伦理原则

15.1 伦理审查

临床试验方案由主要研究者与申办者共同商定，报四川大学华西医院伦理委员会审批后实施。若本方案在临床试验实施过程中进行了修订，需再次报请伦理委员会批准后实施。如发现涉及试验用药的重要新资料则必须将知情同意书作书面修改送伦理委员会批准后，再次取得受试者同意。

15.2 受益与风险

在进入临床研究之前，经动物毒性实验研究未发现明显毒性反应，但你亦有可能会出现不可预知的不良反应。对治疗可能出现的副作用已经制订了医疗对策，包括研究者有权根据自己的判断中止该病例的临床试验。

研究可能为该类疾病的治疗增加一种治疗手段，以用于患有相似病情的其他病人。受试者将在研究期间获得良好的医疗服务。

15.3 招募受试者

招募方式有 2 种：①院内布告的方式；②临床医生（兼研究者）招募。招募受试者布告和研究简介应提交伦理委员会审查。

15.4 知情同意的过程

研究者必须向受试者说明有关临床试验的详细情况，包括试验目的、试验程序、可能的受益和风险、受试者的权利和义务等，使受试者充分理解并有充分的时间考虑、所提问题均得到满意答复后表示同意，并签署“知情同意书”后方能开始临床试验。每一例病人签署知情同意书时医生要将自己的联系电话留给病人，以便病人在出现病情变化时能够随时找到医生。知情同意书提交伦理委员会审查。

15.5 受试者的医疗和保护

研究者负责受试者的医疗，做出与临床试验相关的医疗决定，保证受试者在试验期间出现不良事件时得到适当的治疗。

申办者应与研究者迅速研究所发生的严重不良事件，采取必要的措施以保证受试者的安全和权益，并及时向药品监督管理部门报告，同时向涉及同一药品的临床试验的其他研究者通报不良事件。

申办者对与试验相关的损害或死亡承担治疗的费用及相应的经济补偿。受试者在临床试验期间将免费获得试验药物，免费进行试验理化检查。如果发生与试验药物有关的不良事件，还将得到免费的治疗。

15.6 受试者隐私的保护

只有参与临床试验的研究人员和监查员才可能接触到受试者的个人医疗记录，他们将签署“研究者声明”或“保密承诺”中包括保密内容。药品监督管理部门有权视查临床试验记录。数据处理时将采用“数据匿名”的方式，省略可识别受试者个体身份的信息。受试者的医疗记录将保存在国家药物临床试验机构的资料档案室。

16 伦理学要求

16.1 临床试验必须遵循《赫尔辛基宣言》（2008 年版）和我国有关临床试验研究规范、法规进行。在试验开始之前，试验方案应由临床研究负责单位的伦理委员会批准后方可实施临床试验。

若本方案在临床试验实际执行过程中出现问题，需要对本方案进行修订，请向申请人提出，经多中心协调委员会协商讨论，由负责单位对方案作必要的修订，以书面的形式提交申请人和各参研单位签字认可，再次报请伦理委员会批准后实施。

如发现涉及试验用药品的重要新资料，则必须将知情同意书作书面修改并送伦理委员会批准后，再次取得受试者同意。

16.2 每一位患者入选本研究前，研究医师有责任以书面文字形式，向其或其指定代表人完整、全面地介绍本研究的目的、程序和可能的风险，可供选用的其它治疗方法以及《赫尔辛基宣言》规定的受试者的权利和义务等。试验结果对受试者利大于弊，使受试者监护人充分了解后表示同意，并签署 “知情同意书”后方可开始临床试验。每一例受试者都要留下详细的地址、电话等资料，同时医生要将自己的联系电话留给受试者监护人，以便当受试者出现病情

变化时能够随时找到医生，这也有利于医生随时了解病情变化，提醒患者及时复诊，避免失访。知情同意书应作为临床试验文档保留备查。

17 资料保存

17.1 研究者保存的资料

试验方案及其修正案(已签名)、知情同意书(样式)、伦理委员会批件、伦理委员会成员表、原始门诊或住院病历、病例报告表(已填写、签名、注明日期)、研究者致申办者的严重不良事件报告的原件。

17.2 申办者保存的资料

临床试验申请书、临床前实验资料、国家药品监督管理局批件、研究者履历及研究资格审查表、试验用药品的药检证明、监查报告、新研究者的履历、病例报告表(已填写、签名、注明日期)的原件副本、新批号试验用药的药检证明、申办者致药品监督管理局及伦理委员会的未预期的严重不良反应报告的原件。

17.3 研究者和申办者共同保存的资料

研究者手册、病例报告表(样表)、临床试验协议书(已签名)、临床试验有关的实验室检测正常值范围、试验用药品与试验相关物资的运货单、研究者手册更新件、医学、实验室检查、操作的正常值范围更新，中期或年度报告、研究者签名样张及其他文件(方案、病例报告表、知情同意书、书面情况通知)的更新。

18 各方承担职责与论文发表规定

18.1 申办者、研究者、研究负责单位、参加研究单位均须切实按照药品临床试验管理规范及本方案的规定，承担相应的职责。

18.2 论文发表规定：试验结束后，负责单位有权以论文形式发表本临床试验总结报告，但发表之前，必须征得申办者同意，各研究单位的研究者享有论文署名权。

19 试验结束后的随访和医疗措施

受试者在临床试验期间如果出现不良事件 / 不良反应经处理后，须在半月内随访，以保

证受试者的安全。如果受试者结束试验，但疾病尚未痊愈仍需要治疗，可以采用目前标准的治疗药物进行治疗。

申办者、研究者、监查员均需切实按照《药物临床试验质量管理规范》以及本方案的规定，承担相应的职责。

20 试验总结

20.1 病例报告表验收和原始病历保存。

20.1.1 病例报告表在试验期间由研究者填写后，应立即交本研究负责人验收、保存。

20.1.2 全部病例报告表原件（包括剔除、脱落病例报告表）经监查员和各研究负责人按本方案第 13 项“数据管理”的要求，检查病例和病例报告表的填写，审核签字后交数据管理员。数据管理员负责数据录入，统计分析员负责统计，主要研究者撰写临床试验总结报告，资料存档。试验原始病历由各医院病历室或基地档案室保存。

20.2 揭盲人员与方法

揭盲程序分两级。统计分析前进行第一次揭盲，开出 A、B、C 组。完成统计分析并写出统计分析报告后进行第二次揭盲，开出大剂量组、小剂量组、零剂量（安慰剂）组。揭盲地点：负责单位所在地。参加揭盲盲人员：监查员、负责单位项目负责人、数据管理员、统计负责人。

20.3 总结与小结

统计单位负责建立临床试验数据库，统一进行数据处理，由统计分析人员进行统计，撰写统计分析报告。各参加单位负责完成临床试验中心小结表，签字盖章后交负责单位 1 份存档和申办者若干份。负责单位负责完成清风感咳颗粒“临床试验总结报告”，盖章后交申办者若干份。

21 临床试验预期进度和完成日期

21.1 试验开始时间

方案经伦理委员会批准后，试验药品、研究资料及经费到位之日起开始进行临床试验。

21.2 中期协调会

根据试验进度及完成情况确定是否召开及召开时间。

21.3 完成时间

试验开始后6个月完成。

21.4 试验资料收集、统计及总结时间

试验完成、临床试验数据锁定后，于1个月内完成总结。

22 参考文献

[1] 国家药品监督管理局：《药物临床试验质量管理规范》（局令第3号）2003年；

[2] 国家食品药品监督管理局：《药品注册管理办法》2007年；

[3] 郑筱萸,主编.中药新药临床研究指导原则(试行).第 1 版.北京:中国医药科技出版社,2002年5月

[4] 苏炳华. 新药临床统计分析新进展. 第1版. 上海：上海科学技术文献出版社，2000；

[5] 中医病证诊断疗效标准（中华人民共和国中医药行业标准，国家中医药管理局发布)；

[6] 中华医学会呼吸病学分会哮喘学组. 咳嗽的诊断与治疗指南(2009 版). 中华结核和呼吸杂志.2009,32(6):407-413；

[7] 清风感咳颗粒处方组成，功能主治；供临床医师参阅的药理、毒理结论综述；

[8] 马建岭等.祛风宣肺颗粒治疗感染后咳嗽60例临床观察. 北京中医药大学学报,2007，30（1）:67～69；

[9] DENG Wei-wu.Diagnosis and treatment of chronic cough. Section of Respiratory System of Foreign Medical Sciences,2005, 25(1):70-72；

[10] Braman S S.Postinfectious Cough:ACCP Evidence-Based Clinical Practice Guidelines.chest,2006,129:138S-146S；

[11] Diagnosis and management of cough executive summary.ACCP evidence-based clinical practice guidelines.Chest,2006,129:1S-29S；

[12] LEE PCL,JAWAD M S,ECCLES R.Antitussive efficacy of dextromethorpham in cough associated with acute upper respiratory tract infections.Pham.Pharmacol J,2000,52(9):1137-1142；

附件一 清风感咳颗粒临床试验流程图

阶段	访视期		
就诊	访视 1	访视 2	访视 3
天数	服药前	服药后第 6±1 天	服药后第 11±2 天
**采集基本病史**			
签署知情同意书	×		
填写一般资料	×		
既往病史和治疗史	×		
合并疾病和症状	×		
体格检查	×	×	×
合并用药		×	×
**有效性观察**			
咳嗽临床症状评分	×	×	×
中医证候积分	×	×	×
CQLQ 量表	×		×
**安全性观察**			
血常规、尿常规	×		×
肝、肾功能	×		×
心电图	×		×
**排除性观察**			
胸片	×		
支气管激发或舒张试验	×		
尿妊娠实验（育龄妇女）	×		
**不良事件观察**			
不良事件记录		×	×
**其它工作**			
随机分组	×		
分发药物		×	
药物数量统计		×	×

附件二 清风感咳颗粒临床研究受试者须知

尊敬的受试者：

经你的医生检查，你已确诊为感染后咳嗽（风邪恋肺证），我们将邀请你参加清风感咳颗粒治疗感染后咳嗽（风邪恋肺证）的临床研究。该药物是包头中药有限责任公司研制的中药新药，已获得国家食品药品监督管理局临床研究批件（批件号：2010L00279）。此临床研究是对清风感咳颗粒治疗感染后咳嗽（风邪恋肺证）有效性和安全性作出初步评价。

1. 研究背景

清风感咳颗粒是在多年临床经验基础上总结出的用之于感染后咳嗽的临床有效方药，由麻黄、青风藤、百部、紫菀组成，功能主治为宣肺祛风、止咳化痰。用于风邪恋肺证，症见咳嗽、咽痒、痰黏而少、咽干、咳嗽常因吸入冷热空气或刺激性气味诱发加重、胸闷，感染后咳嗽见上述症侯者。

临床前药效学实验显示，清风感咳颗粒具有镇咳、祛痰、平喘、抗炎和免疫功能抑制作用。动物急毒、长毒实验研究显示，清风感咳颗粒按临床推荐剂量使用，毒副作用小，是安全可靠的，同时也不引起延迟性毒副反应。

按照国家中药新药临床研究指导原则的临床试验要求，受包头中药有限责任公司和北京岐黄药品临床研究中心委托，由四川大学华西医院为负责单位，另外四家医院为参加单位，对清风感咳颗粒治疗感染后咳嗽（风邪恋肺证）有效性和安全性作初步评价。

2. 研究概述

采用区组随机对照，多中心临床试验方法，由四川大学华西医院负责与其它四所医院（辽宁中医药大学附属医院、南京市第一医院、包头市中心医院、第四军医大学唐都医院）协同完成。本临床研究预计有 180 例受试者自愿参加，试验药物为清风感咳颗粒，对照药物为清风感咳颗粒安慰剂。如果您符合入选标准并愿意参加本研究，您将被随机分配到试验组或对照组的任一组，服药方法为：清风感咳颗粒 6g/次+清风感咳颗粒安慰剂 6g/次，2 次/日，温水冲服；或清风感咳颗粒 12g /次，2 次/日，温水冲服；或清风感咳颗粒安慰剂 12g /次，2 次/日，温水冲服。试验时间为 10 天。

试验前研究者将完成您的病例登记、体格检查、心电图、血常规、小便常规、肝肾功能、胸片、支气管激发或舒张试验等各项实验室指标检查，还需耽误您几分钟时间完善 CQLQ 量表。服药后第 6 天、第 11 天观察记录一般情况、临床症状和体征。请您在服药结束后 1 天内，空腹来医院复查。医生将对您疾病的改善及实验室检测指标进行检查。研究期间请您按照医生的要求服药，按时复诊，并随时告知医生您的不适感受。研究期间，请您保证不服用其他治疗咳嗽的药物，如有其他合并用药请在访视时告知医生。

3. 不良反应

动物急毒、长毒实验研究显示，按临床推荐剂量使用，毒副作用小，是安全可靠的，同时也不引起延迟性毒副反应。

4. 可能的受益和风险

自愿参加本研究，你将得到免费的药物（本试验仅供 10 天，试验结束后不再供药）及相关的实验室检查，将可能使您的疾病得到改善。试验结束后，你还将获得申办方提供的交通补贴。

临床前实验研究虽未发现明显毒副反应，但你亦有可能会出现不可预知的不良反应。我们将认真检测你有可能发生的不良反应，如果在临床试验期间，出现因药物引起的已知的或目前未知的不良反应，医生均会给予你积极的治疗，申办单位将会负责由此引起的相关治疗费用。如果发生与受试药物相关的严重不良事件，除医生会给予积极治疗外，申办单位将会承担治疗费用和相应的赔偿。

5. 保密

本临床试验的结果只用于科研目的，因此您参加试验及试验中您的个人资料均属保密，将依照法律规定得到保护，不会泄露您的名字和身份，您的姓名不会出现在任何研究报告和公开出版物中。但国家食品药品监督管理局、华西医院伦理委员会、研究者、申办者或监察员等如因工作需要，按规定有权接触您所有的试验资料，包括临床试验观察表、实验数据等。

6. 自愿参加

本临床试验遵循《药物临床试验质量管理规范》和《赫尔辛基宣言》，并获得临床负责单位四川大学华西医院伦理委员会的审核、批准，方案设计合乎伦理要求，这将会保证您的权益在本试验中不受侵犯。

您参加本临床试验完全是自愿的，您可以拒绝参加或在任何时间退出试验，而不会遭到歧视或报复，您的医疗待遇与权益也不会受影响。如果您退出临床试验，为了安全考虑，您在退出时应该完成一些相应的医学检查。另外，如果研究者认为您不适合继续参加时，为保护您的利益，研究者可以决定您是否继续参加临床试验。退出试验的受试者会得到研究者有关下一步治疗的建议。

清风感咳颗粒临床试验受试者知情同意

本人已仔细阅读**“<u>清风感咳颗粒临床研究受试者须知</u>”**，已了解这是一项治疗感染后咳嗽（风邪恋肺证）药物有效性与安全性初步评价的临床研究，临床试验研究者已就此药的特点和可能存在的不良反应向我做了详细解释，并对有关问题给予了解答。我在充分了解受试者须知的全部内容以及参加受试带来的利益与风险后，自愿参加本试验。我已充分理解：

1、此项研究已经获得国家食品药品监督管理局批准（批件号：2010L00279）；

2、作为受试者，我将遵守本研究受试者须知要求；

3、本临床试验的结果用于科研目的，除外国家食品药品监督管理局、华西医院伦理委员会或申办单位等，我参加试验及试验中的个人资料均属保密，将依照法律规定得到保护；

4、我自愿参加本研究，如果在临床试验期间，出现因药物引起的已知的或目前未知的不良反应（包括发生与受试药物相关的严重不良事件），医生均会给予我积极的治疗，申办单位将会负责由此引起的相关治疗费用。如发生与受试药物相关的严重不良事件，我还会得到申办单位的相应赔偿；

5、我参加本临床试验完全是自愿的，我可以拒绝参加或在任何时间退出试验，而不会遭到歧视或报复，我的医疗待遇与权益亦不会受影响；

6、我自愿参加本试验，并与研究人员充分合作；

7、我保证如实、客观地向研究人员提供参加本研究前的健康状况及相关情况。

受试者签名：____________________　　研究者签名：______________________

联系电话：______________________　　联系电话：________________________

日期：______年______月______日　　日期：_______年______月_______日

附件三 药物性肝病处理参考预案（SOP）

药物性肝病（drug induced liver disease）是由于药物或/及代谢产物引起的肝脏损害。

1、药物性肝病分类

药物性肝病的临床表现不一，根据损肝药物的种类及引起肝病的机制不同分为：

⑴急性肝细胞损害：此类最为多见。

①以肝细胞坏死为主，常有发热、乏力、纳差和血清转氨酶升高（正常 2-30 倍），ALP 和白蛋白受影响较小（ALT/ALP≥5），高胆红素血症和凝血酶原时间延长与肝损害严重程度相关。重者发生爆发性肝功能衰竭，出现进行性黄疸、出血倾向和肝性脑病，常发生死亡。

②以过敏反应为主的急性药物性肝病，常有发热、皮疹、黄疸、淋巴结肿大，伴血清转氨酶、胆红素和 ALP 中度升高，药物接触史常较短（<4 周）。

③以胆汁淤积为主的药物性肝病，与肝内淤胆、肝外胆道梗阻、急性胆管炎相似，临床表现有发热、黄疸、上腹痛、骚痒、右上腹压痛及肝肿大，并伴有血清转氨酶轻度升高、ALP 明显升高（2-10 倍）、胆盐、脂蛋白 X、GGT 及胆固醇升高。一般停药后 3 月～3 年恢复，少数可发展为肝硬化。

⑵慢性肝细胞损害：可以轻到无症状，而重到发生伴肝性脑病的肝功能衰竭。生化表现有血清转氨酶、G-GT 的升高，进展型可导致肝硬化伴低蛋白血症及凝血功能障碍。

2、药物性肝病处理

诊断药物性肝病时，应该了解：⑴用药史；⑵是否有基础肝病；⑶以往有无药物过敏史或过敏性疾病史。一旦诊断药物性肝损害，可按照以下处理进行：

⑴停用导致药物性肝病或可能引起药物性肝病的药物；

⑵适当休息，加强营养，支持疗法，给予高蛋白、高糖低脂饮食，补充维生素 C、B 和 E。

⑶腺苷胆氨酸（思美泰）：1-2g/d 静滴 2 周，以后改为 1.6g/d 分 2 次口服，直到症状及生化指标改善，一般为 4-8 周。

⑷肝得健：1～2 粒/次，1 日 3 次；注射液 2 支，1 日 1 次，重症病例可 2～4 支/日。

⑸重症患者导致肝功能衰竭或重度胆汁淤积，进展到肝硬化时，内科治疗无效时，应联系外科，考虑肝移植。

附件四 药物性肾损害处理参考预案（SOP）

药物性肾损害是由于药物或/及代谢产物引起的肾脏损害。由于临床药物的广泛应用，药物所致的肾脏损害日益受到人们的重视。研究显示，药物所致的肾脏损害发生率呈上升趋势。不同的药物所致的肾脏损害，其机制、损伤部位、临床表现都不尽相同。

1、药物性肾损害分类

药物性肾损害的临床表现不一，根据引起肾脏损害的机制不同可分为：

⑴剂量依赖性肾损害

肾脏损害的程度与投药量成比例，损害的部位以近曲小管为主。代表药物为抗癌药、氨基糖甙类抗生素。

⑵非剂量依赖性肾损害

此类肾损害与投药量无关。目前普遍认为其机制是人体激素/体液或细胞介导的免疫反应，组织学上呈现间质性肾炎。以青霉素类药物引起的肾损害为代表。

⑶血流动力学机制引起的肾损害

肾小球的出入球动张力受前列腺素、血管紧张素的调节。某些药物通过影响这些内源性因素改变肾脏的血流动力学而导致肾损害，也可通过影响肾小球血管对血管收缩和扩张的敏感性而致血流动力学异常。

⑷其他

虽然将药物性肾损害的机制分为 3 种，但事实上药物导致肾损害既可通过单一机制致病，也可几种机制同时致病。如 NSAIDs 既通过剂量依赖性机制引起肾小管病变和通过非剂量依赖性机制引发间质性病变，又通过对肾小球出、入小动脉的作用，而引发肾小球病变。

2、药物性肾损害的处理

诊断药物性肾损害时，应该了解：⑴用药史；⑵是否存在可能产生药物性肾损害的各种危险因素，如：老龄、基础肾脏疾病、糖尿病、各种可能引起肾灌注不足的情况等。⑶以往有无药物过敏史或过敏性疾病史。一旦诊断药物性肾损害，可按照以下处理进行：

⑴停用导致药物性肾损害或可能引起药物性肾损害的药物。

⑵密切观察尿量和肾损伤指标。密切监测各种肾损害指标,密切观察尿量的变化。

⑶针对药物性肾损害的不同类型, 进行对症处理和对因治疗。

如有可能, 应水化和碱化尿液，努力争取机会增加药物的排出。过敏性急性间质性肾炎可应用皮质激素治疗。对药物引起的急、慢性肾功能衰竭及其并发症, 要积极进行综合治疗或抢救, 必要时进行透析治疗。发生严重急慢性肾衰竭时,可以肾替代治疗。

附件五 咳嗽特异性生活质量问卷（CQLQ）

1	家人和/或亲密的朋友再也不能忍受我咳嗽			
	1.极不相符	2.不相符	3.符合	4.十分符合
2	我长时间缺席一些重要的活动，如：上班、上学或志愿者服务			
	1.极不相符	2.不相符	3.符合	4.十分符合
3	我完全不能参加一些重要的活动，如：上班、上学或志愿者服务			
	1.极不相符	2.不相符	3.符合	4.十分符合
4	我没有食欲			
	1.极不相符	2.不相符	3.符合	4.十分符合
5	我因咳嗽感到胃部不适及呕吐			
	1.极不相符	2.不相符	3.符合	4.十分符合
6	我因咳嗽而干呕			
	1.极不相符	2.不相符	3.符合	4.十分符合
7	我担心我可能得了艾滋病或结核			
	1.极不相符	2.不相符	3.符合	4.十分符合
8	我感到头痛			
	1.极不相符	2.不相符	3.符合	4.十分符合
9	我担心自己得了癌症			
	1.极不相符	2.不相符	3.符合	4.十分符合
10	我头昏			
	1.极不相符	2.不相符	3.符合	4.十分符合
11	我因咳嗽而尿湿裤子			
	1.极不相符	2.不相符	3.符合	4.十分符合
12	我因咳嗽大便弄脏裤子			
	1.极不相符	2.不相符	3.符合	4.十分符合
13	我因咳嗽而出汗			
	1.极不相符	2.不相符	3.符合	4.十分符合
14	我因咳嗽而声嘶			
	1.极不相符	2.不相符	3.符合	4.十分符合
15	我呼吸的时候感到胸痛或腹痛			
	1.极不相符	2.不相符	3.符合	4.十分符合
16	我因咳嗽而致肋骨骨折			
	1.极不相符	2.不相符	3.符合	4.十分符合
17	我因咳嗽而致夜间不能入睡			
	1.极不相符	2.不相符	3.符合	4.十分符合
18	我因咳嗽而中断电话交谈			
	1.极不相符	2.不相符	3.符合	4.十分符合
19	我因咳嗽而不能再唱歌，如在教室里			

	1.极不相符	2.不相符	3.符合	4.十分符合
20	我已经停止参加一些社会活动，如：看电影、比赛和会议			
	1.极不相符	2.不相符	3.符合	4.十分符合
21	我不得不改变我的生活方式			
	1.极不相符	2.不相符	3.符合	4.十分符合
22	我全身疼痛			
	1.极不相符	2.不相符	3.符合	4.十分符合
23	我因咳嗽而感到疲倦乏力			
	1.极不相符	2.不相符	3.符合	4.十分符合
24	我因咳嗽而感到尴尬难看			
	1.极不相符	2.不相符	3.符合	4.十分符合
25	我因别人认为我有问题而感到不安			
	1.极不相符	2.不相符	3.符合	4.十分符合
26	我想再次确认我没有严重问题			
	1.极不相符	2.不相符	3.符合	4.十分符合
27	我因咳嗽而感到不自然或害羞			
	1.极不相符	2.不相符	3.符合	4.十分符合
28	我担心我可能有很严重的问题			
	1.极不相符	2.不相符	3.符合	4.十分符合

附件六 用药包装标签

大盒标签式样：

药物编号：

清风感咳颗粒临床研究用药

（仅供临床研究用）

［包 装 规 格］每大盒内装 4 小盒。
［用 法 用 量］详见小盒标签。
［试 验 疗 程］10 天。
［有 效 期］至 2012 年 8 月
［药 物 批 号］100901,100902
［贮 存］置于儿童不易触及处。密封，置阴凉干燥处保存。
［注 意］剩余药物和包装请务必交还医生。

包头中药有限责任公司

小盒标签式样：

药物编号：

清风感咳颗粒临床研究用药

（仅供临床研究用）

［包 装 规 格］每小盒内装 12 袋，每袋内装 6 克。
［功 能 主 治］用于风邪恋肺证，症见咳嗽、痰粘而少、咽痒、咽干、咳嗽常因吸入冷热空气或刺激性气味诱发加重、胸闷，感染后咳嗽见上述症侯者。
［用 法 用 量］一次 2 袋，一日 2 次，温开水冲服。
［试 验 疗 程］10 天。
［有 效 期］至 2012 年 8 月
［药 物 批 号］100901,100902
［贮 存］置于儿童不易触及处。密封，置阴凉干燥处保存。
［注 意］剩余药物和包装请务必交还医生。

包头中药有限责任公司

附件七 支气管激发试验操作规程

通过定量雾化吸入乙酰甲胆碱，诱发气道收缩反应，以肺功能指标第一秒用力呼气容积（FEV_1)的改变来判定气道缩窄的程度,并以FEV_1较基础值下降20%时的累积激发药物剂量(PD_{20} FEV_1）来表示气道反应性。

一. 试剂

激发剂采用乙酰甲胆碱，以生理盐水稀释配制为水溶液。容器上标明浓度和配制时间（具体浓度配置见测定步骤3），置冰箱低温（4℃）保存。使用前需从冰箱取出并在室温下放置30min。

二. 测试仪器

（一） 肺量计：需符合美国胸科协会（ATS）与欧洲呼吸学会（ERS）技术标准。使用时每天均需经标准 3.00L 定标筒进行标化，流量误差应小于±3%。打印定标报告并存档。

（二)雾化装置:采用定量雾化器,其雾化质量应达到要求,雾化颗粒的直径大小应<5μ m,释雾量恒定。常用装置可参见表 1。

表 1 常用定量雾化吸入装置

编号	仪器名称	图片
1	德国 Jaeger 公司 APS (Aerosol Provocation System)定量雾化吸入装置	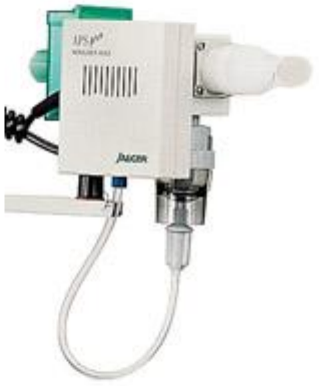
2	比利时 Medisoft 公司一体化计算机控制喷药装置	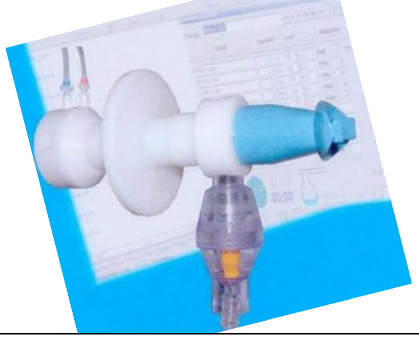

3	Devilbiss N045 手捏式雾化器	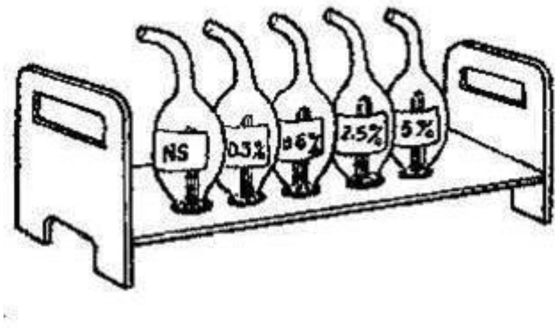

（三）测定步骤（图 1）

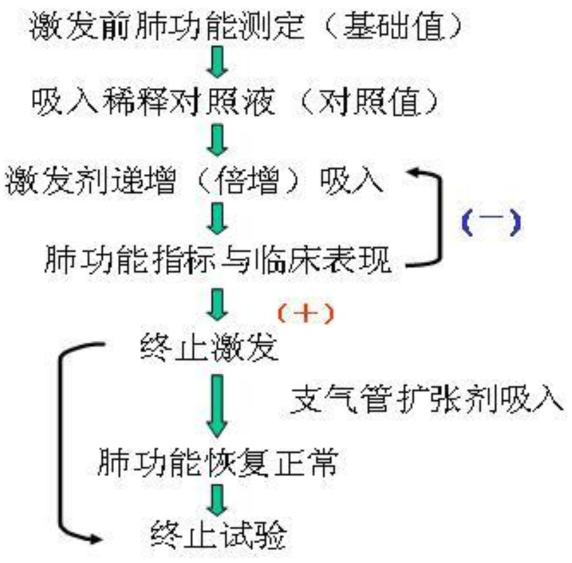

图1 激发试验程序

1.测定基础肺功能。受试者休息 15 分钟，取坐位，夹鼻，先测定 FEV_1 基础值，取最高值。测定方法要求达到 ATS/ERS 用力肺活量质量控制标准。主要包括：①试验起始标准：外推容量＜5%FVC 或 150ml（取较大值）；②试验结束标准：呼气时间≥6 秒（10 岁以上的受检者），或时间-容量曲线显示持续 1 秒以上容量变化＜25ml；③可重复性标准：至少测定 3 次，FVC、FEV_1 最佳值与次佳值之间差异＜150ml。

2.吸入生理盐水，再测定肺功能。一方面，让患者认识吸入激发药物的过程，减轻其心理负担，熟悉吸入方法，增加吸入过程的协从性；另一方面，观察稀释液生理盐水是否对肺通气功能有所影响，作为吸入激发物的对照。若吸入生理盐水后 FEV_1 下降＜10%，可继续下一步试验；若 FEV_1 下降＞10%，则其本身即可增加气道反应性，或患者经数次深吸气诱发气道痉挛，其气道反应性较高，此时需从最低剂量开始吸入激发药物，并严密观察，谨慎进行，同

时在试验报告中注明。

3.吸入乙酰甲胆碱，再测定肺功能。从低剂量开始，双倍递增，吸入后再测定肺功能，直至 FEV_1 较基础值下降≥20％，或出现明显的不适及临床症状，或吸入最高剂量为止。对基础肺功能正常，无喘息病史的受试者，可适当从较高剂量（0.40μ mol 或 0.08mg）开始，或采用 4 倍递增的简化程序吸入激发药物，但当 FEV_1 较基础值下降≥10%时，应恢复为原 2 倍递增方式继续吸入。

不同定量吸入装置的设置或有差别，各中心可根据吸入装置配制不同浓度的激发药物，**但需保证患者累计吸入乙酰甲胆碱最高剂量为 12.8μ mol（2.5mg），且能够计算 PD20-FEV1。**表 2 和表 3 分别列举了 APS 定量雾化吸入法(Jaeger 肺功能仪专用)和简易手捏式雾化吸入法的吸药顺序以供参考。

表 2 APS 定量雾化吸入法吸药顺序和剂量（乙酰甲胆碱）

激发液浓度	常规程序		简化程序		累积剂量	
	顺序	单次剂量	顺序	单次剂量	mg	μ mol
-	R1	-	R1	-		-
0.9%NaCl	R2		R2			
4mg/ml	P1	0.0098mg			0.0098	0.05
4mg/ml	P2	0.0098mg			0.0196	0.10
4mg/ml	P3	0.0196mg			0.039	0.20
4mg/ml	P4	0.039mg	P1	0.078mg	0.078	0.40
32mg/ml	P5	0.078mg			0.156	0.80
32mg/ml	P6	0.156mg	P2	0.235mg	0.313	1.60
32mg/ml	P7	0.313mg			0.626	3.20
32mg/ml	P8	0.626mg	P3	0.939mg	1.252	6.40
32mg/ml	P9	1.252mg	P4	1.252mg	2.504	12.80
沙丁胺醇	D	200~400μ g	D			

表 3 简易手捏式雾化吸入法吸药顺序和剂量（乙酰甲胆碱）

顺序	常规程序（2 倍递增）		简化程序（4 倍递增）		累积剂量	
	浓度	喷药次数	浓度	喷药次数	mg	μ mol
1	0.3%	1			0.010	0.05
2	0.3%	1			0.020	0.10
3	0.6%	1			0.039	0.20
4	0.6%	2	0.6%	4	0.078	0.40
5	2.5%	1			0.156	0.80
6	2.5%	2	2.5%	3	0.313	1.60
7	2.5%	4			0.626	3.20
8	5.0%	4	5%	6	1.252	6.40
9	5.0%	8	5%	8	2.504	12.80

4.结果报告

①定性判断标准：在试验过程中，当 FEV_1 较基础值下降≥20%时，可判断为激发试验阳性。若吸入最大浓度后，仍未达上述标准，则为激发试验阴性。FEV_1 计算方法如下：

$$FEV_1\text{改变率}(\%)=\frac{\text{激发后}FEV_1\text{检查值}-FEV_1\text{基础值}}{FEV_1\text{基础值}}\times 100\%$$

②定量判断标准：以 FEV_1 较基础值下降 20%时的累积激发药物剂量（PD_{20} FEV_1）来判断，计算方法如下：

$$PD_{20}=\text{antilog}\left[\frac{(\log D_2-\log D_1)\times(20-R_1)}{(R_2-R_1)}+\log D_1\right]$$

D_1＝使 FEV_1 下降 20％前的累积剂量，D_2＝使 FEV_1 下降 20％后的累积剂量，

R_1＝D_1 剂量时的 FEV_1 改变率，R_2＝D_2 剂量时的 FEV_1 改变率。

四、注意事项

（一）禁忌症： FEV_1＜70%预计值；近期心肌梗死、严重心律失常或脑血管意外；未经

控制的高血压；主动脉瘤；上呼吸道感染（<2 周）；妊娠；哮喘发作期。

（二）试验前应嘱患者停用可能干扰检查结果的药物：吸入性短效β_2受体兴奋剂或抗胆碱能药停用 4～6 小时；口服短效β_2受体兴奋剂或茶碱停 8 小时，长效或缓释型停用 24 小时以上；抗组胺药停用 48～72 小时；色甘酸钠停用 24 小时；糖皮质激素口服停 48 小时，吸入停 12 小时。

参考文献

1. ATS. Guidelines for Methacholine and Exercise Challenge Testing. Am J Respir Crit Care Med 2000;161:309-329.

2. MR Miler,J Hankinson,V Brusasco,et al.Standardisation of spirometry. Eur Respir J 2005;26:319～338.

3. 中华医学会编著.临床技术操作规范.呼吸病学分册.北京:人民军医出版社，2008:62-68.
