# Supplementary figures and images for: The efficacy of QingfengGanke granule in treating postinfectious cough in pathogenic wind invading lungs syndrome: a multicenter, randomized, double-blind, placebo-controlled trial
Source: Chin Med. 2015 Aug 9;10:21. doi: 10.1186/s13020-015-0049-6 (PMC4529711; doi:10.1186/s13020-015-0049-6)

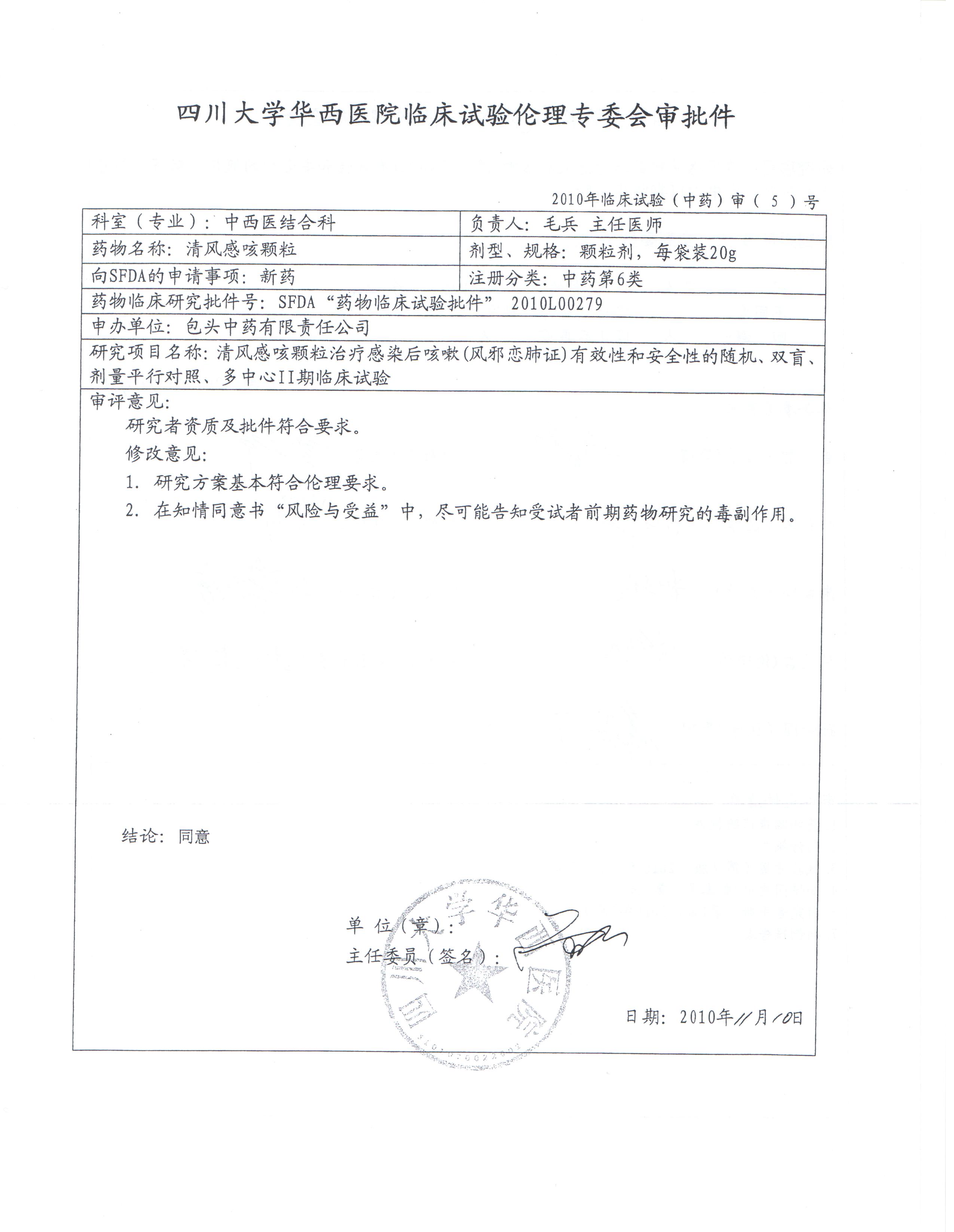

Supplement: Additional file 3. — Approval of ethics committee. [file 13020_2015_49_MOESM3_ESM.jpeg]
