# Supplementary material for: The efficacy of QingfengGanke granule in treating postinfectious cough in pathogenic wind invading lungs syndrome: a multicenter, randomized, double-blind, placebo-controlled trial
Source: Chin Med. 2015 Aug 9;10:21. doi: 10.1186/s13020-015-0049-6 (PMC4529711; doi:10.1186/s13020-015-0049-6)
Supplement: Supplementary file 4 [file 13020_2015_49_MOESM4_ESM.doc]

**CHECKLIST
ITEMS**

**Extension Combination :**   Consort    Herbal medicine interventions

**Item\_1 : TITLE
& ABSTRACT**

|  |  |  |
| --- | --- | --- |
|  |  | **Reported on Page N°** |
| **Consort** | **1a : Identification as a randomised trial in the title  1b : Structured summary of trial design, methods, results, and conclusions** | **1**  **3-4** |
| **Herbal medicine interventions** | Either the title or abstract, or both, should state the herbal medicinal product's Latin binomial, the part of the plant used, and the type of prepara | NA |

**Item\_2 :
INTRODUCTION Background and objectives**

|  |  |  |
| --- | --- | --- |
|  |  | **Reported on Page N°** |
| **Consort** | **2a : Scientific background and explanation of rationale  2b : Specific objectives or hypotheses** | **5**  **6** |
| **Herbal medicine interventions** | 2a: Including a brief statement of reasons for the trial with reference to the specific herbal medicinal product being tested and, if applicable, whether new or traditional indications are being investigated | 5 |

**Item\_3 : METHODS
Trial design**

|  |  |  |
| --- | --- | --- |
|  |  | **Reported on Page N°** |
| **Consort** | **3a : Description of trial design (such as parallel, factorial) including allocation ratio  3b : Important changes to methods after trial commencement (such as eligibility criteria), with reasons** | **6**  **NA** |

**Item\_4 : METHODS
Participants**

|  |  |  |
| --- | --- | --- |
|  |  | **Reported on Page N°** |
| **Consort** | **4a : Eligibility criteria for participants  4b : Settings and locations where the data were collected** | **22**  **7** |
| **Herbal medicine interventions** | If a traditional indication is being tested, a description of how the traditional theories and concepts were maintained. For example, participant inclusion criteria should reflect the theories and concepts underlying the traditional indication | 7 |

**Item\_5 : METHODS
Interventions**

|  |  |  |
| --- | --- | --- |
|  |  | **Reported on Page N°** |
| **Consort** | **5 : The interventions for each group with sufficient details to allow replication, including how and when they were actually administered** | **7** |
| **Herbal medicine interventions** | Where applicable, the description of an herbal intervention should include:   5a: Herbal medicinal product name:  1. The Latin binomial name together with botanical authority and family name for each herbal ingredient; common name(s) should also be included  2. The proprietary product name (i.e. brand name) or the extract name (e.g. EGb-761) and the name of the manufacturer of the product  3. Whether the product used is authorised (licensed, registered) in the country in which the study was conducted  5b: Characteristics of the herbal product:  1. The part(s) of plant used to produce the product or extract  2. The type of product used (e.g. raw [fresh or dry], extract)  3. The type and concentration of extraction solvent used (e.g. 80% ethanol, 100% H2O, 90% glycerine, etc.) and the ratio of herbal drug to extract (e.g. 2 to 1)  4. The method of authentication of raw material (i.e. how done and by whom) and the lot number of the raw material. State if a voucher specimen (i.e. retention sample) was retained and, if so, where it is kept or deposited, and the reference number  5c: Dosage regimen and quantitative description:  1. The dosage of the product, the duration of administration, and how these were determined  2. The content (e.g. as weight, concentration; may be given as range where appropriate) of all quantified herbal product constituents, both native and added, per dosage unit form. Added materials, such as binders, fillers, and other excipients (e.g. 17% maltodextrin, 3% silicon dioxide per capsule), should also be listed  3. For standardized products, the quantity of active/marker constituents per dosage unit form  5d: Qualitative testing:  1. Products chemical fingerprint and methods used (equipment and chemical reference standards) and who performed the chemical analysis (e.g. the name of the laboratory used); whether a sample of the product (i.e. retention sample) was retained and if so, where it is kept or deposited  2. Description of any special testing/purity testing (e.g. heavy metal or other contaminant testing) undertaken, which unwanted components were removed and how (i.e. methods)  3. Standardisation: what to standardise (e.g. which chemical components of the product) and how (e.g. chemical processes or biological/functional measures of activity)  5e: Placebo/control group: The rationale for the type of control/placebo used   5f: Practitioner: a description of the practitioners (e.g. training and practice experience) that are a part of the intervention | 5  21 |

**Item\_6 : METHODS
Outcomes**

|  |  |  |
| --- | --- | --- |
|  |  | **Reported on Page N°** |
| **Consort** | **6a : Completely defined pre-specified primary and secondary outcome measures, including how and when they were assessed  6b : Any changes to trial outcomes after the trial commenced, with reasons** | **8-9**  **NA** |
| **Herbal medicine interventions** | Outcome measures should reflect the intervention and indications tested considering, where applicable, underlying theories and concepts | 8-9 |

**Item\_7 : METHODS
Sample size**

|  |  |  |
| --- | --- | --- |
|  |  | **Reported on Page N°** |
| **Consort** | **7a : How sample size was determined  7b : When applicable, explanation of any interim analyses and stopping guidelines** | **9** |

**Item\_8 : METHODS
Randomisation : Sequence generation**

|  |  |  |
| --- | --- | --- |
|  |  | **Reported on Page N°** |
| **Consort** | **8a : Method used to generate the random allocation sequence  8b : Type of randomisation; details of any restriction (such as blocking and block size)** | **6**  **6** |

**Item\_9 : METHODS
Randomisation : Allocation concealment mechanism**

|  |  |  |
| --- | --- | --- |
|  |  | **Reported on Page N°** |
| **Consort** | **9 : Mechanism used to implement the random allocation sequence (such as sequentially numbered containers), describing any steps taken to conceal the sequence until interventions were assigned** | **6** |

**Item\_10 :
METHODS Implementation**

|  |  |  |
| --- | --- | --- |
|  |  | **Reported on Page N°** |
| **Consort** | **10 : Who generated the random allocation sequence, who enrolled participants, and who assigned participants to interventions** | **6,7,8** |

**Item\_11 :
METHODS Blinding**

|  |  |  |
| --- | --- | --- |
|  |  | **Reported on Page N°** |
| **Consort** | **11a : If done, who was blinded after assignment to interventions (for example, participants, care providers, those assessing outcomes) and how  11b : If relevant, description of the similarity of interventions** | **6,7** |

**Item\_12 :
METHODS Statistical methods**

|  |  |  |
| --- | --- | --- |
|  |  | **Reported on Page N°** |
| **Consort** | **12a : Statistical methods used to compare groups for primary and secondary outcomes  12b : Methods for additional analyses, such as subgroup analyses and adjusted analyses** | **10** |

**Item\_13 :
RESULTS Participant flow (a diagram is strongly recommended)**

|  |  |  |
| --- | --- | --- |
|  |  | **Reported on Page N°** |
| **Consort** | **13a : For each group, the numbers of participants who were randomly assigned, received intended treatment, and were analysed for the primary outcome  13b : For each group, losses and exclusions after randomisation, together with reasons** | **10**  **10** |

**Item\_14 :
RESULTS Recruitment**

|  |  |  |
| --- | --- | --- |
|  |  | **Reported on Page N°** |
| **Consort** | **14a : Dates defining the periods of recruitment and follow-up  14b : Why the trial ended or was stopped** | **10** |

**Item\_15 :
RESULTS Baseline data**

|  |  |  |
| --- | --- | --- |
|  |  | **Reported on Page N°** |
| **Consort** | **15 : A table showing baseline demographic and clinical characteristics for each group** | **22** |
| **Herbal medicine interventions** | Including concomitant medications, herbal and complementary medicine use | 11 |

**Item\_16 :
RESULTS Numbers analysed**

|  |  |  |
| --- | --- | --- |
|  |  | **Reported on Page N°** |
| **Consort** | **16 : For each group, number of participants (denominator) included in each analysis and whether the analysis was by original assigned groups** | **10** |

**Item\_17 :
RESULTS Outcomes and estimation**

|  |  |  |
| --- | --- | --- |
|  |  | **Reported on Page N°** |
| **Consort** | **17a : For each primary and secondary outcome, results for each group, and the estimated effect size and its precision (such as 95% confidence interval)  17b : For binary outcomes, presentation of both absolute and relative effect sizes is recommended** | **11,12** |

**Item\_18 :
RESULTS Ancillary analyses**

|  |  |  |
| --- | --- | --- |
|  |  | **Reported on Page N°** |
| **Consort** | **18 : Results of any other analyses performed, including subgroup analyses and adjusted analyses, distinguishing pre-specified from exploratory** | **NA** |

**Item\_19 :
RESULTS Harms**

|  |  |  |
| --- | --- | --- |
|  |  | **Reported on Page N°** |
| **Consort** | **19 : All important harms or unintended effects in each group** | **13** |

**Item\_20 :
DISCUSSION Limitations**

|  |  |  |
| --- | --- | --- |
|  |  | **Reported on Page N°** |
| **Consort** | **20 : Trial limitations; addressing sources of potential bias, imprecision, and, if relevant, multiplicity of analyses** | **15,16** |

**Item\_21 :
DISCUSSION Generalisability**

|  |  |  |
| --- | --- | --- |
|  |  | **Reported on Page N°** |
| **Consort** | **21 : Generalisability (external validity, applicability) of the trial findings** | **14,15** |
| **Herbal medicine interventions** | Where possible, discuss how the herbal product and dosage regimen used relate to what is used in self-care and/or practice | NA |

**Item\_22 :
DISCUSSION Interpretation**

|  |  |  |
| --- | --- | --- |
|  |  | **Reported on Page N°** |
| **Consort** | **22 : Interpretation consistent with results, balancing benefits and harms, and considering other relevant evidence** | **14,15** |
| **Herbal medicine interventions** | Interpretation of the results in light of the product and dosage regimen used. Discussion of the trial results in relation to the trials of other available products | 14,15 |

**Item\_23 : OTHER
INFORMATION Registration**

|  |  |  |
| --- | --- | --- |
|  |  | **Reported on Page N°** |
| **Consort** | **23 : Registration number and name of trial registry** | **4** |

**Item\_24 : OTHER
INFORMATION Protocol**

|  |  |  |
| --- | --- | --- |
|  |  | **Reported on Page N°** |
| **Consort** | **24 : Where the full trial protocol can be accessed, if available** | **6** |

**Item\_25 : OTHER
INFORMATION Funding**

|  |  |  |
| --- | --- | --- |
|  |  | **Reported on Page N°** |
| **Consort** | **25 : Sources of funding and other support (such as supply of drugs), role of funders** | **17** |

|  |
| --- |
| **REFERENCES :** |
| **Consort** | Schulz KF, Altman DG, Moher D, for the CONSORT Group. CONSORT 2010 Statement: updated guidelines for reporting parallel group randomised trials. Ann Int Med 2010;152.    Moher D, Hopewell S, Schulz KF, Montori V, Gtzsche PC, Devereaux PJ, Elbourne D, Egger M, Altman DG, for the CONSORT Group. CONSORT 2010 Explanation and Elaboration: updated guidelines for reporting parallel group randomised trial. BMJ 2010; 340: c869. |
| **Herbal medicine interventions** | Gagnier JJ, Boon H, Rochon P, Moher D, Barnes J, Bombardier C. Reporting randomized, controlled trials of herbal interventions: an elaborated CONSORT statement. Ann Intern Med 2006; 144(5):364-367.     Gagnier JJ, Boon H, Rochon P, Moher D, Barnes J, Bombardier C. Recommendations for reporting randomized controlled trials of herbal interventions: explanation and elaboration. J Clin Epidemiol 2006; 59(11):1134-1149. |
